# A three-component model of the spinal nerve ramification: Bringing together the human gross anatomy and modern Embryology

**DOI:** 10.3389/fnins.2022.1009542

**Published:** 2023-01-16

**Authors:** Shunsaku Homma, Takako Shimada, Ikuo Wada, Katsuji Kumaki, Noboru Sato, Hiroyuki Yaginuma

**Affiliations:** ^1^Department of Neuroanatomy and Embryology, Fukushima Medical University, Fukushima, Japan; ^2^Department of Cell Science, Institute of Biomedical Sciences, Fukushima Medical University, Fukushima, Japan; ^3^Division of Gross Anatomy and Morphogenesis, Graduate School of Medical and Dental Sciences, Niigata University, Niigata, Japan

**Keywords:** primaxial/abaxial, epaxial/hypaxial, motor neuron, Lhx3, FoxP1, mesoderm, connective tissue

## Abstract

Due to its long history, the study of human gross anatomy has not adequately incorporated modern embryological findings; consequently, the current understanding has often been incompatible with recent discoveries from molecular studies. Notably, the traditional epaxial and hypaxial muscle distinction, and their corresponding innervation by the dorsal and ventral rami of the spinal nerve, do not correspond to the primaxial and abaxial muscle distinction, defined by the mesodermal lineages of target tissues. To resolve the disagreement between adult anatomy and embryology, we here propose a novel hypothetical model of spinal nerve ramification. Our model is based on the previously unknown developmental process of the intercostal nerves. Observations of these nerves in the mouse embryos revealed that the intercostal nerves initially had superficial and deep ventral branches, which is contrary to the general perception of a single ventral branch. The initial dual innervation pattern later changes into an adult-like single branch pattern following the retraction of the superficial branch. The modified intercostal nerves consist of the canonical ventral branches and novel branches that run on the muscular surface of the thorax, which sprout from the lateral cutaneous branches. We formulated the embryonic branching pattern into the hypothetical ramification model of the human spinal nerve so that the branching pattern is compatible with the developmental context of the target muscles. In our model, every spinal nerve consists of three components: (1) segmental branches that innervate the primaxial muscles, including the dorsal rami, and short branches and long superficial anterior branches from the ventral rami; (2) plexus-forming intramural branches, the serial homolog of the canonical intercostal nerves, which innervate the abaxial portion of the body wall; and (3) plexus-forming extramural branches, the series of novel branches located outside of the body wall, which innervate the girdle and limb muscles. The selective elaboration or deletion of each component successfully explains the reasoning for the standard morphology and variability of the spinal nerve. Therefore, our model brings a novel understanding of spinal nerve development and valuable information for basic and clinical sciences regarding the diverse branching patterns of the spinal nerve.

## Introduction

The branching pattern of the spinal nerve constitutes an essential chapter of the human gross anatomy and has long been a critical topic in basic and clinical medicine. Therefore, it may seem that there is nothing new to discover or define regarding the gross anatomy of the spinal nerve. However, conventional concepts in gross anatomy have not caught up with recent progress in the study of molecular embryology. As a result, using adult morphology for analysis makes it difficult to accurately describe embryonic phenomena. Two significant issues exist concerning the use of conventional terms for embryological studies.

The first issue is that the traditional muscle classification based on the adult anatomy of the spinal nerve is incompatible with the embryonic lineages of the target muscles. The conventional epaxial/hypaxial anatomy for muscle classification is based on the differential innervation of the two primary rami of the spinal nerve; the dorsal and ventral rami. The dorsal rami innervate the intrinsic back muscles, whereas the ventral rami innervate the other body wall, girdle and limb muscles (Spörle, 2001; Standring, 2015). However, this conventional classification is incompatible with primaxial/abaxial embryological classification, derived from the developmental concept of the patterning domains in the vertebrate body (Burke and Nowicki, 2003). Moreover, the conventional epaxial and hypaxial dichotomy does not adequately reflect the patterning events during development (Burke and Nowicki, 2003).

The second issue regarding the use of adult morphology for embryonic analysis is the types of target muscles that correspond to the motor columns of the spinal cord. The spinal motor neurons (MNs) are organized in a columnar fashion. This organization was initially prescribed based on the locations of a specific motor neuron group to innervate a particular muscle (motor neuron pool) within the ventral horn (Callister et al., 1987; Gutman et al., 1993; Kitamura and Richmond, 1994; Vanderhorst and Holstege, 1997; Watson et al., 2009). Later, the combinatorial expression patterns of the LIM-class homeobox transcription factors coincide with the columnar organization (Tsuchida et al., 1994; Jessell, 2000; Dasen et al., 2008). For this reason, the current nomenclature of the motor columns has evolved independently of the knowledge gained from molecular studies on MN diversity, leading to several proposals of muscle types that correspond to the motor column.

Accordingly, to bridge the gap between adult anatomy and modern embryology, we propose a novel hypothetical spinal nerve ramification model based on our findings on mouse intercostal nerve development. Our model provides an anatomical foundation for embryonic research and a never-described comprehensive theory for the standard and variable branching patterns of the spinal nerve.

## Results

### Problems around the spinal nerve with the mesodermal tissue lineages

**FIGURE 1:**
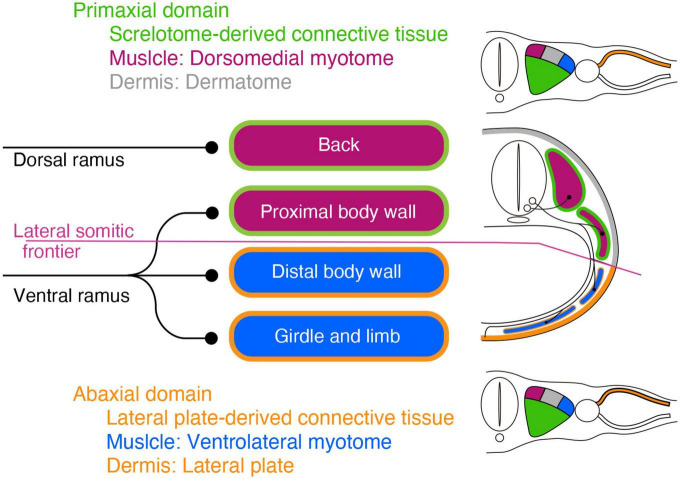
Schematic illustration of the primaxial and abaxial muscles and their innervations by the dorsal and ventral rami. Primaxial muscle progenitors derive from the dorsomedial myotome (plum-color) and differentiate into the back and proximal body wall muscles within the sclerotome-derived connective tissue (green) environment. Abaxial muscle progenitors are from the ventrolateral myotome (blue) and differentiate into the distal body wall, girdle, and limb muscles within the lateral plate-derived connective tissue environment (oak). The primaxial and abaxial domains are mutually exclusive, and there is a front line, the lateral somitic frontier (amethyst-color), between them. The two main branches, the dorsal and ventral rami, do not fully correspond to the embryonic domains. The dorsal rami innervate the back muscles in the primaxial domain, whereas the ventral rami innervate both the proximal body wall muscles in the primaxial domain, as well as the distal body wall muscles, girdle and limb muscles in the abaxial domain. The dermatome and its-derived primaxial dermis are colored gray. The primaxial dermis derived from the lateral plate mesoderm is colored oak.

The target tissues of the spinal nerve, such as the dermis and muscles, are mosaics of cells derived from various portions of the mesoderm (Figure 1; Ordahl and Le Douarin, 1992; Olivera-Martinez et al., 2000; Bothe et al., 2007; Christ et al., 2007). Two different lineages of connective tissues demarcate the vertebrate body into two global environments (Burke and Nowicki, 2003; Winslow et al., 2007). The term “primaxial” describes the sclerotome-derived connective tissue environment, whereas “abaxial” characterizes the lateral plate mesoderm-derived connective tissue environment. The lateral somitic frontier is the boundary between the primaxial and abaxial domains. The muscle progenitors derived from the dorsomedial portion of the somite differentiate into the back muscles and proximal body wall muscles within the primaxial domain. In contrast, those from the ventrolateral part differentiate into the distal body wall muscles as well as the girdle and limb muscles exclusively in the abaxial domain (Ordahl and Le Douarin, 1992; Olivera-Martinez et al., 2000).

Conceptually, the embryonic primaxial/abaxial distinction divides the body wall into proximal and distal regions Liem and Aoyama, 2009;. Therefore, the embryonic primaxial/abaxial distinction does not fully correspond to the traditional classification of epaxial/hypaxial muscles, which is based on the differential innervation of the two primary rami of the spinal nerve, dorsal and ventral rami. The dorsal rami innervate the intrinsic back muscles, whereas the ventral rami innervate the body wall, girdle and limb muscles (Spörle, 2001; Standring, 2015). As a result, the two embryonic domains are not functional with the two adult primary branches; the ventral rami of the spinal nerve innervate all of the abaxial muscles and some of the primaxial muscles, whereas the dorsal rami exclusively innervate the rest of the primaxial muscles. Moreover, the conventional epaxial and hypaxial dichotomy does not adequately reflect the patterning events during development (Burke and Nowicki, 2003). To deal with these irregularities, we currently employ the primaxial-abaxial and epaxial-hypaxial distinctions as two concepts with different respective purposes; one for describing developmental phenomena, and the other for describing adult anatomy (Burke and Nowicki, 2003; Gilbert and Barresi, 2016). However, this does not necessarily indicate that the spinal nerve branching pattern is independent of the embryonic muscle lineages. Because the spinal nerve does not fall under the primaxial-abaxial definition, the connection between the spinal nerve ramification and muscle lineages cannot be known until an analysis is performed.

As the muscles have two different lineages, the dermis also has two separate embryonic origins (Figure 1; Mauger, 1972; Fliniaux et al., 2004; Durland et al., 2008). The dermis in the primaxial and abaxial domains are from the somite and the LPM, respectively. The dual sources of the dermis raise an additional issue of how the spinal nerve establishes the coherent innervation to the primaxial and abaxial portions in a single sensory dermatome in the body wall.

Another issue is how the columnar organization of spinal motor neurons can be constructed in a manner compatible with the primaxial and abaxial lineages of the target muscles. The MNs in the spinal cord are organized into two primary columns, the lateral motor column (LMC) and the medial motor column (MMC), with the latter further subdivided into the medial (MMCm) and lateral (MMCl) portions. Our conventional understanding is that MMCm MNs innervate the epaxial muscles via the dorsal rami, whereas the MMCl and LMC MNs innervate the hypaxial muscles via the ventral rami (Callister et al., 1987; Gutman et al., 1993; Kitamura and Richmond, 1994; Vanderhorst and Holstege, 1997; Watson et al., 2009). However, the analysis of maker expression patterns in the motor pool revealed that some motor pools in the MMCm send axons through the ventral rami (Stillhard, 1981; Luxenhofer et al., 2014; Nagashima et al., 2020). These erratic innervation patterns imply that we may need to conciliate between the MMC MNs and their target muscles.

### The intercostal nerve displayed a dual innervation pattern to the ventral midline region during the early developmental period, but later changed into the single adult form

To the best of our knowledge, there have been no reports to date of a compelling link between the adult spinal nerve morphology and mesodermal lineages of the target muscles. Therefore, we decided to reappraise the development of the spinal nerve. Interestingly, the mouse embryo in Figure 1, from the paper by Hua et al. (2013) and the modified micrograph from that Figure on the front cover of the book Developmental Biology by Gilbert and Barresi (2016) exhibited two seemingly equivalent branches of intercostal nerves (ICNs) toward the ventral midline region. This configuration of the ICNs contradicts the general perception of the ICNs, which have only one anterior (ventral) branch. The dual ventral branches of the ICNs led us to suspect that the spinal nerve might develop differently than we have generally understood.

**FIGURE 2:**
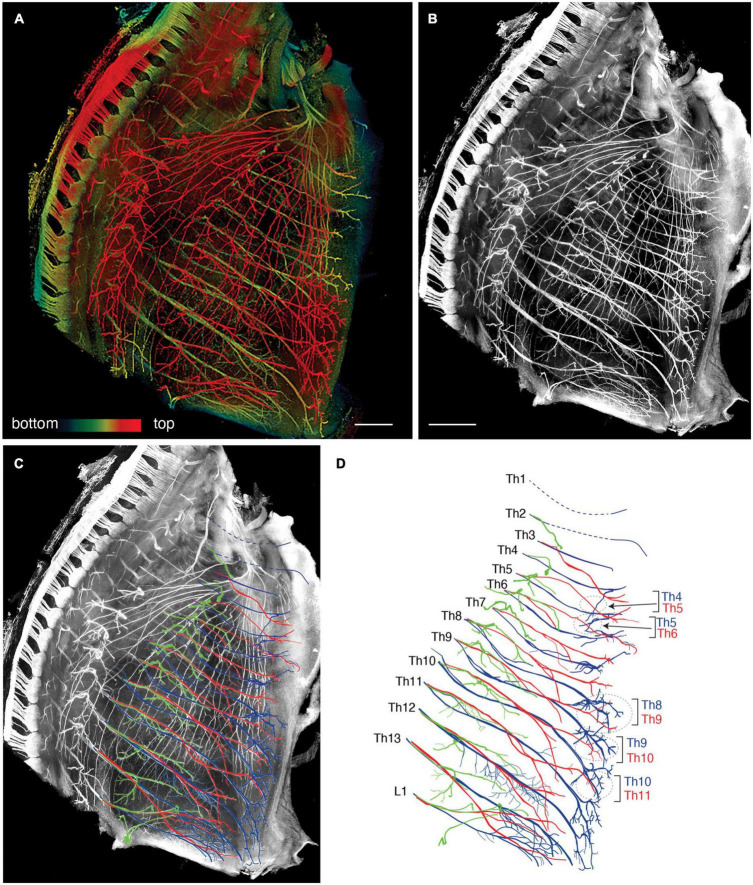
Branching pattern of the intercostal nerve in a transparent thoracic wall from a mouse embryo on E13. All micrographs are the unilateral side of the thoracic wall, and are in surface view. The dorsal side is toward the left of the micrographs. The anterior side is toward the top of the micrographs. **(A)** MIP image. Nerve branches are color-coded; those close to the outer surface are colored red, and those close to the parietal surface are blue. **(B)** A micrograph of a black and white MIP image without depth information. The vertically running branches are for the dermal panniculus muscle from the brachial plexus (Petruska et al., 2014). **(C)** Traced intercostal nerves; the deep branches (blue), superficial branches (red), and lateral cutaneous branches (green). When the surface skin was peeled off, the terminal branches of the lateral cutaneous branches were also removed. No tracing of the branches for the dermal muscle is included. **(D)** Illustration of the traced branches. The deep branches are in blue; the superficial branches are in red; and the lateral cutaneous branches are in green. The hatched circles indicate overlapping innervations by the nearby superficial and deep branches.

We reanalyzed the development of the ICNs immunohistochemically using whole-mount transparent specimens of the thoracic walls from mouse embryos. As demonstrated in the previous study, the ICNs bifurcated into the superficial and deep branches in the thoracic walls of mouse embryos on embryonic day (E) 13 (Figure 2). The lateral cutaneous branches (LCBs) diverged from the superficial branches (Figure 2).

**FIGURE 3:**
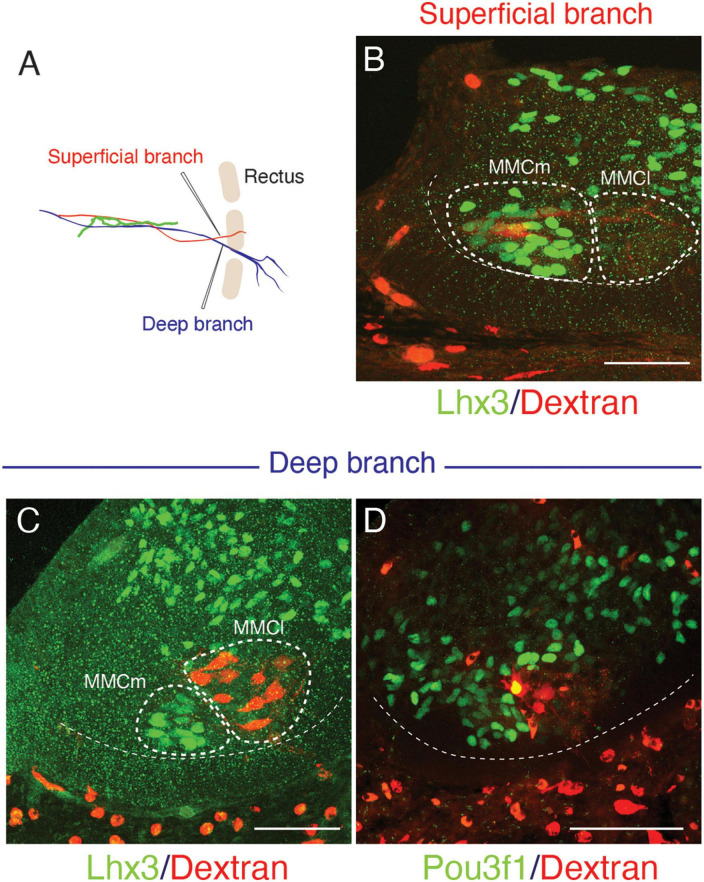
Differential origins of the superficial and deep branches of the intercostal nerves. All micrographs are transverse sections of the lower thoracic spinal cord from E13 embryos. The margins of the ventral horns are in thin dashed lines. The midline of the spinal cord is toward the left side, and the dorsal side is toward the upside of the micrographs. The thick dashed lines encircle the medial (MMCm), and lateral (MMCl) halves of the medial motor columns. **(A)** The injection points are in the superficial (red) and deep (blue) branches, which are close to the rectus muscle band (light brown). **(B)** The labeling (red) is in the Lhx3-positive (green nuclei) motor neurons, which are in the MMCm, but not in the MMCl following the injection into the superficial branches. **(C,D)** Following injection into the deep branches, labeling is restricted within the MMCl, where Lhx3 expression is negative, and the Pou3f1-positive nuclei (green) with a high expression level are observed in the labeled motor neurons (red) in the MMCl.

The superficial branch from an axial level and the deep branch above or below the axial level often innervated the same areas around the ventral midline. The overlapping innervation by spinal nerves from the two nearby axial levels indicates that the superficial and deep branches may receive different axial specifications. Hence, one branch is not merely the collateral branch from the other; instead, they are likely to be distinctive from each other. The current back-tracing study supports this possibility. We injected a tracer into the superficial and deep branches of the lowest four ICNs of the E13 embryos as closely as possible to the putative location of the rectus muscle. The injection into the superficial branches resulted in labeling the MNs that express the MMCm marker Lhx3, in the thoracic spinal cord (Figures 3A, B). Following injection into the deep branches, the labeled MNs were Lhx3-negative in MMCl (Figures 3A, C). Some labeled MNs expressed Pou3f1 (Oct6, SCIP), a marker for phrenic MNs (Figure 3D; Rousso et al., 2008). We have never observed labeled MNs in the MMCm following injection into the deep branches. Thus, the two equal-looking ventral branches of the ICNs are from different motor columns, demonstrating a previously unrecognized ICN branching pattern of the existence of the two different ventral (anterior) branches.

**FIGURE 4:**
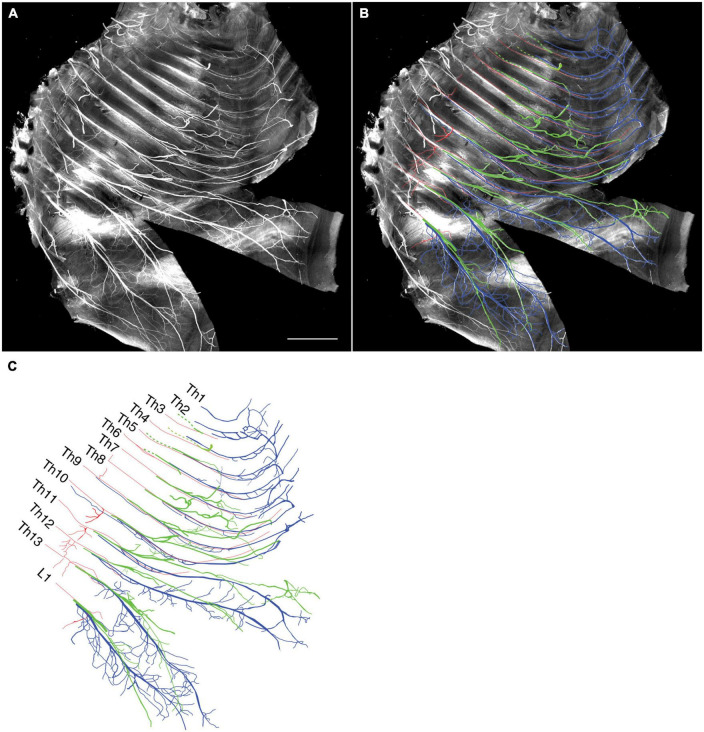
Branching pattern of the intercostal nerve from a mouse embryo on E15. All micrographs are in the surface view of the unilateral side of the thoracic wall from an E15 embryo. The dorsal side is toward the left of the micrographs. The anterior side is toward the top of the micrographs. Because of the pronounced curvature of the thoracic wall, the two split lines are in the lower portion of the wall for mounting. **(A)** MIP image. **(B,C)** Tracing of the branches. The deep branch is in blue, the superficial branch is in red, and the lateral cutaneous branch is in green.

**FIGURE 5:**
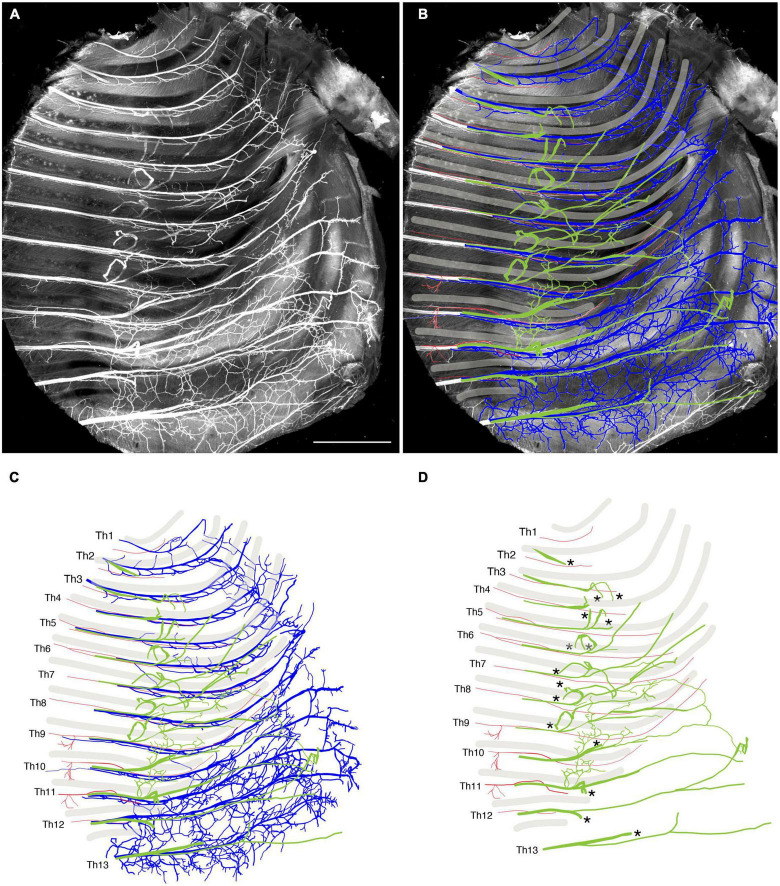
Close-up view of the thoracic wall from an E15 embryo. The anterior side is toward the top of the micrographs. The costal side is toward the right of the micrographs. The cartilaginous costae are indicated by gray bars. **(A)** Black and white MIP image. **(B,C)** The superficial branches (red) run along the lower margin of the costae while the deep (blue) and lateral cutaneous (green) branches change their courses toward one or more of the above intercostal spaces. **(D)** The superficial and lateral cutaneous branches are extracted for comparison, showing the segmental and non-meristic innervation patterns of the superficial and lateral cutaneous branches, respectively. Asterisks are stumps of the dermal branches of the lateral cutaneous branch, because the skin and dermis were removed for transparency.

We tracked the developmental course of the ICNs. The branching patterns in the slightly older (E15) embryos were substantially different from those in the E13 embryos (Figures 4, 5). The thick superficial branch was no longer observed in the ICNs of the E15 embryos; fine meristic branches were observed with the termination shortly before the ventral midline in the superficial portion of the thoracic wall. It was unclear that the fine superficial branches were remnants of the degenerated superficial branches, or the muscular branches destined for the external intercostal muscles. On the other hand, the deep branches traveled along the lower margins of the costae toward the anterior midline, where the terminal branches that originated from different axial levels communicated. The deep branches from the lower axial levels took similar trajectories to those of the human thoracolumbar nerves by deviating from the original intercostal space.

Peeling off the dermis together with the LCBs for better clarity revealed novel branches that stemmed out from the root of the LCBs and ran ventrally on the muscular surface of the thoracic wall (Figures 4, 5). These novel branches from the LCBs changed their running directions around the middle of the costae. They traveled along an intercostal space and then turned, moving anteriorly by one or more intercostal space, often uniting with neighboring novel branches.

Collectively, our observations of the high-resolution deep imaging of the embryonic ICNs unraveled previously undiscovered developmental features of the ICNs: (1) the ICNs initially have two distinctive ventral (anterior) branches from the MMCm and MMCl; (2) superficial ventral branches from the MMCm degenerate; consequently, the overall branching pattern becomes the widely-perceived adult morphology; and (3) novel branches diverged from the LCBs develop on the muscular surface of the thoracic wall.

### Bisecting the spinal nerve’s ramification into the segmental and plexus-forming components may eliminate the difficulty of reconciling the adult anatomy with embryonic domains

Although the branches from the ventral rami are involved in a plexus formation while the dorsal rami maintain a segmental pattern, there is a series of non-plexus-forming branches that are diverged from the root segment of the ventral ramus of every spinal nerve. We found that the difficulties coordinating the adult anatomy with embryonic patterning domains begin with these segmental branches from the ventral rami. The muscles that receive segmental branches from the ventral rami include: the longus colli and scalenus in the cervical region; the rhomboid, levator scapulae, and serratus anterior in the brachial region; the serratus posterior (superior and inferior) in the thoracic region; and the quadratus lumborum in the lumbar region. However, the segmental branches to the rhomboid, levator scapulae, and serratus anterior in the brachial region do not maintain individuality, merging into the dorsal scapula or long thoracic nerves.

The muscles listed above fall into specific muscle classification categories, initially described by Nishi (Supplementary Table 1; Nishi, 1938; Sato, 1968). Moreover, these muscles are reported to belong exclusively to the primaxial domain and, thus, are inserted into primaxial portions of the bones (Burke and Nowicki, 2003; Durland et al., 2008). We believe that these apparent coincidences are likely to have occurred not by chance, but because the factor in determining the points from which the branches diverge reflects the spatial and temporal factors of the nerve-muscle interactions during development.

**FIGURE 6:**
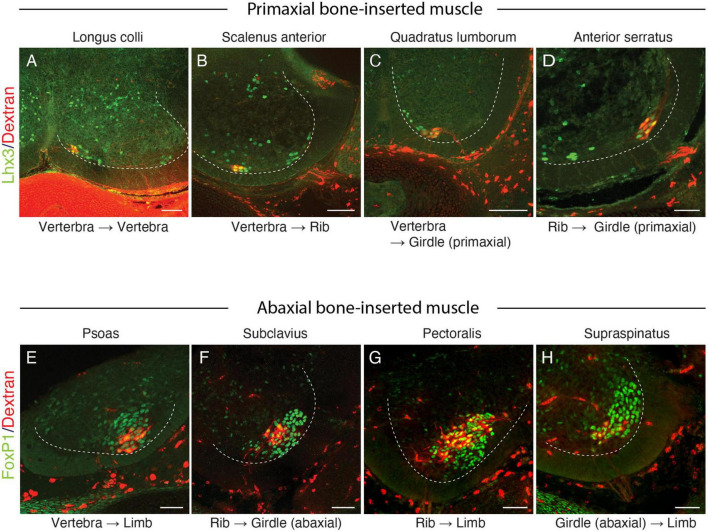
Lhx3 and FoxP1 expressions in MNs innervating muscles spanning various skeletal elements. Lhx3 expression **(A–D)** (green nuclei) and FoxP1 (**E,H**, green nuclei) in labeled MNs (red) following the injection of the tracer (Alexa594-labeled dextran) into the target muscles. The ventral quadrant of the brachial and lumbar spinal cord from E13 **(A–C,E–H)** or E15 **(D)** mouse embryos are shown, and the dashed lines mark the ventral horns. In all micrographs, the midline and the dorsal side are located toward the left and top of the image, respectively. Scale bars, 100 μm.

The current study also demonstrates that the parental MNs for some of the muscles listed above, longus colli, scalenus anterior, serratus anterior, and quadratus lumborum, are Lhx3-positive (Figures 6A–D). Previous studies have also demonstrated that Lhx3-positive MNs innervate the rhomboid, anterior serratus muscles (Tsuchida et al., 1994; Nagashima et al., 2020), and the longus colli (Luxenhofer et al., 2014). The location of the labeled MNs for the serratus anterior is unique: it is in the lateral side of the ventral horn, compared to the other MNs, which are located in the medial side.

The dorsal rami, which innervate the intrinsic back muscles, are segmental. The motor origin of the dorsal rami is also Lhx3-positive (Tsuchida et al., 1994; Agalliu et al., 2009). Therefore, the examined muscles in the above list share the gross morphological innervation pattern (segmental) and molecular profile of the parental MNs (Lhx3 expression) with those of the dorsal rami. Accordingly, we hypothesize that the segmental branches originating from the Lhx3-positive MNs innervate the muscles (or muscle portions) within the primaxial domain. By categorizing the segmental branches into the primaxial muscle-innervating ones, our hypothesis successfully integrates the dorsal rami and the minor segmental branches from the ventral rami.

The nature of the branches to the external intercostal nerve meets our criteria of the branch for the primaxial muscle. The branches to the external intercostal muscles are distinctive branches segmentally diverged from the main trunk of the ICNs (Maurer, 1905; Satoh, 1969). Moreover, Lhx3-positive MNs innervate the external intercostal muscles (Nagashima et al., 2020). Therefore, the external intercostal muscles are primaxial in our model. In the human anatomy, the external intercostal muscles occupy the proximal intercostal spaces, whereas the inner and innermost intercostal muscles reside in the distal intercostal spaces (Standring, 2015). Consistent with this, the proximal and distal intercostal spaces are primaxial and abaxial, respectively (Durland et al., 2008).

The current study also demonstrates that the superficial ventral branches in the mouse embryo originate from the Lhx3-positive MNs. For this reason, we categorized the superficial anterior branches as a primaxial muscle-innervating branch.

**FIGURE 7:**
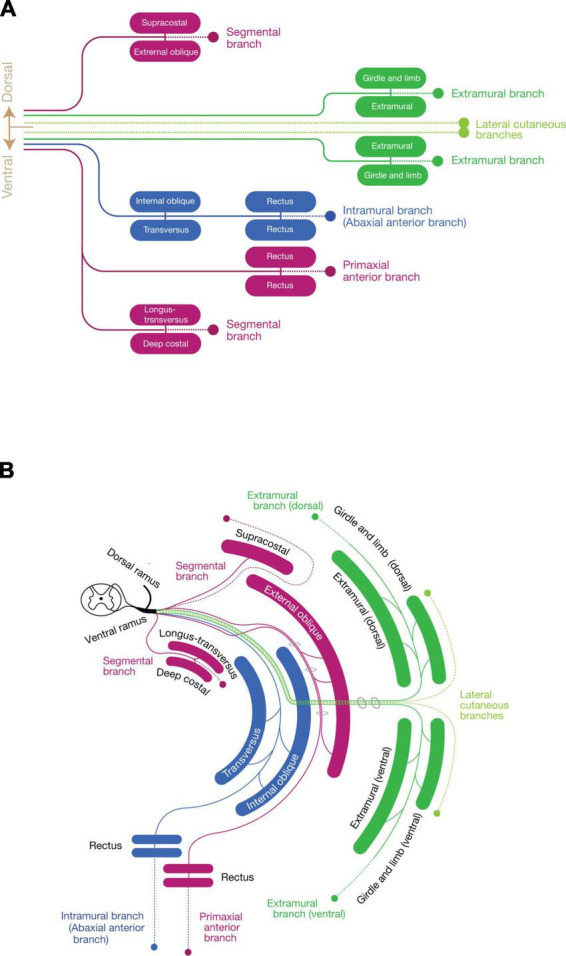
Schematic depiction of the three components mapped to the muscle classes. **(A)** Map of the three nerve components to muscle classes. Primaxial muscle-innervating branches (purple) include the dorsal segmental branches to the supracostal and external oblique, the ventral segmental branches to the longus-transversus and deep costal muscles classes, and the primaxial anterior branch to the primaxial rectus class. The LCB (light green) also consists of a pair of the dorsal and ventral subbranches, accompanying the dorsal and ventral extramural branches (dark green) to the girdle and limb muscle classes. The intramural branch (blue) innervates the internal oblique, transversus, and rectus muscle classes, and no dorsal counterpart exists. **(B)** Diagram with the layer information. The terminal branches of the three-components run in different inter-muscular spaces: the primaxial anterior branch in the superficial inter-muscular space, the intramural branch in the deep inter-muscular space, and the extramural branch in the outside of the body wall. A basic building unit is created from the innervation pattern of the dorsal rami to the four subclasses of the intrinsic back muscles; one main branch sends muscle-innervating twigs to a parallel pair of muscles, and ends as one terminal sensory branch (broken lines). Note that the body wall is composed of the eight muscle layers.

Collectively, the spinal nerve radiates the branches to innervate the primaxial muscles (or muscle portion), including the dorsal rami, and segmental branches, together with the primaxial anterior branches from the ventral rami. The dorsal rami no longer stand for the specific branches for the epaxial muscles, but represent the most prominent segmental branches among those which innervate the primaxial muscles (Figures 7A, B).

### Novel understanding of the ICNs: The ICNs have two anterior terminal branches, contrary to the general perception that the ICN ends with a single anterior branch

In this section, based on our embryonic observations, we formulate the hypothesis of the dual innervation of the anterior branches to the muscles within the body wall by taking examples of the normal and anomalous innervations of the human ICNs to the external intercostal and rectus abdominis muscles.

Previously, Kodama (1986) found that a muscle group, including the external intercostal muscles, serratus posterior muscles, and some anomalous supra costal muscles, was consistently innervated by the distinctive segmental branches. These segmental branches were reported to travel through the superficial inter-muscular spaces between the internal and external intercostal muscles from the root segment of the ICNs; hence, they have been named the superficial ICNs. Kodama (1986) also reported that the superficial ICNs reached the anterior midline region in the middle thoracic region, terminating as cutaneous branches. Moreover, the branches even sometimes innervated the rectus abdominis muscle. However, this innervation pattern is paradoxical; the superficial ICNs, responsible for the innervation of the dorsal muscle subgroup (Supplementary Table 1), innervate the rectus muscle, the presumed ventral subgroup muscle (Supplementary Table 1) in the anterior midline region.

**FIGURE 8:**
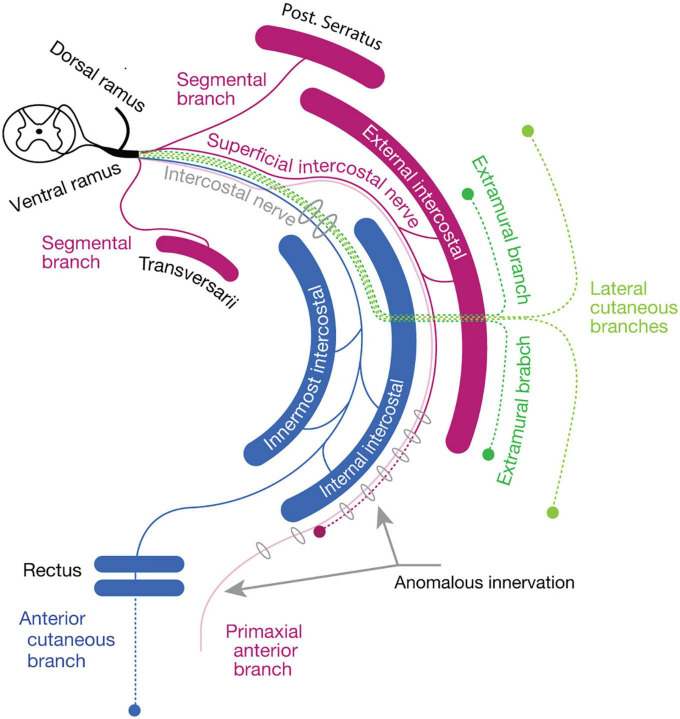
The reasoning for the genesis of an anomalous dual innervation to the rectus muscle. The color code is as same as Figure 7. The anomalous branch, as a vestigial superficial anterior branch (primaxial anterior branch, opaque purple), runs together with the superficial intercostal nerve, creating an appearance of the extension of the superficial intercostal nerve to the rectus muscle.

To resolve this paradoxical innervation, we interpret the above aberrant branch to the rectus abdominus muscle as a remnant of the superficial anterior branch following embryonic modification. Because the superficial anterior branch of the ICNs change the trajectory from the deep intermuscular spaces into the superficial intermuscular spaces on the way to the anterior midline, they may merge with the superficial ICNs (Figure 7B). As demonstrated in our embryonic observations in mouse embryos, the superficial anterior branches of the ICNs in human may also degenerate at the thoracic level during development, leaving only the muscular branches to the external intercostal muscles in the superficial intermuscular spaces (the superficial ICN; Figure 8). Under the anomalous situation, the retraction of the superficial anterior branches of the ICNs is incomplete, and vestigial fibers remain together with the superficial ICNs, thus creating the appearance of the dual innervation of the anterior midline region (Figure 8).

Based on the embryonic innervation pattern of the ICNs and a possible reasoning for the anomalous dual innervation pattern to the human rectus muscles, we hypothesize that every spinal nerve has two anterior branches: the superficial anterior branch in the superficial inter-muscular space and the deep anterior branch in the deep inter-muscular space (Figures 7A, B). Because the parental MNs of the superficial ventral branches are Lhx3-postive in the mouse embryo, the human counterpart, the superficial anterior branches are responsible for the innervation of the primaxial muscles, hence the name “primaxial anterior branch” in our model. On the other hand, the MNs that send out the intercostal nerves are located in the MMCl, and Lhx3- and Foxp1 (a marker for LMC MNs)-negative (Tsuchida et al., 1994; Dasen et al., 2008). Our back-tracing analysis also showed that the intercostal nerves originate from the Lhx3-negative MNs (Figure 3C). We designated the deep anterior branches as a serial homolog of the canonical ICN, namely the intramural branches, which are responsible for the innervation of the abaxial body wall (Figures 7A, B). Entanglement of the intramural branches with the superficial anterior branches forms the plexuses in the cervical and sacral regions.

### Novel class of abaxial domain-innervating branch, the extramural branch

We introduce another class of plexus-forming branches, extramural branches, which contribute to the formation of the brachial and lumbar plexus, and innervate the girdle and limb muscles, which are located outside of the body wall.

Our embryonic observation also revealed novel branches located on the outer surface of the thoracic wall. These surface branches have not been reported so far in the mouse embryo. However, previous human dissection studies reported seemingly equivalent nerve branches on the thoracic and abdominal walls, namely extramural branches (Koizumi and Horiguchi, 1992).

Their studies identified unique branches that diverged from the LCBs on the fascial surface of the body wall. The LCBs of the second to fourth ICN send out minute branches on the fascial surface of the thoracic wall (Koizumi and Horiguchi, 1992). The external oblique muscle is similarly innervated by twigs from the LCBs from the fascial (outer) surface in the lower thoracic levels (Sato, 1973; Schlenz et al., 1999). The LCB from the Th2 (the intercostobrachial nerve) occasionally sends out distinctive branches, which join the pectoral nerve (Koizumi and Horiguchi, 1992). Moreover, the intercostobrachial nerve joins the brachial plexus, suggesting that the most posterior constituent of the brachial plexus is the LCB. From these innervation patterns, the LCBs may be considered a mainframe of the brachial plexus and convey the distinctive nerve component to target muscles outside the body wall, as Cave (1929) suggested. Accordingly, we hypothesized a series of distinct branches, namely the extramural branches, which are accompanied by the LCBs and innervate the muscles in the girdle and limb portions of the abaxial domain, all along the body axis (Figures 7A, B).

The MNs that innervate the girdle and limb muscles are in the LMC and express FoxP1 transcriptional factor (Dasen et al., 2008). Our current back-tracing study also showed that the girdle muscles that are inserted into the abaxial bones, such as the subclavius, pectoralis, supraspinatus, and psoas muscles, are innervated by FoxP1-postive MNs (Figures 6E–H).

### Three component model

In summary (Figure 7), our model states that every single spinal nerve consists of three nerve components: (1) primaxial muscle-innervating branches, which include the segmental branches and primaxial anterior branches radiating from the root segment of the ventral rami, and the dorsal rami; (2) the intramural branch, a homologous branch to the canonical intercostal nerve bound for the innervation of the abaxial muscles within the body wall; and (3) the extramural branch, which travels along the LCB to the abaxial muscles outside the body wall, such as the girdle and limb muscles.

The selective expansion or deletion of each component successfully explains the reasoning for the standard ramification patterns of the spinal nerve at different axial levels. The axial difference in the entanglement of the extra- and intramural branches creates varieties of the nerve plexuses along the body axis. The extramural branches elaborate into the plexus with the backbone of the LCBs, as the regional expansions of the abaxial domains develop into the limb. The intramural branches create the cervical and sacral plexuses to innervate the abaxial body wall, where the muscles, especially the rectus muscles, are specialized for unique functions: the infrahyoid muscles for swallowing in the cervical region; and the sphincter muscles for defecation and urination in the sacral region. At the thoracic level, the intramural branches remain segmental as the ICNs, possibly because of the spatial constraint created by the costae.

The target muscles of the three components are in Supplementary Table 1.

### Detailed extrapolation of our model to the human body

We here illustrate the peripheral innervation pattern of the spinal nerve based on our model in detail. All major branches of the spinal nerve are described as one of the three components in our model. The target muscle classification is described in the Supplementary Information (Supplementary Table 1).

#### Thoracic region

**FIGURE 9:**
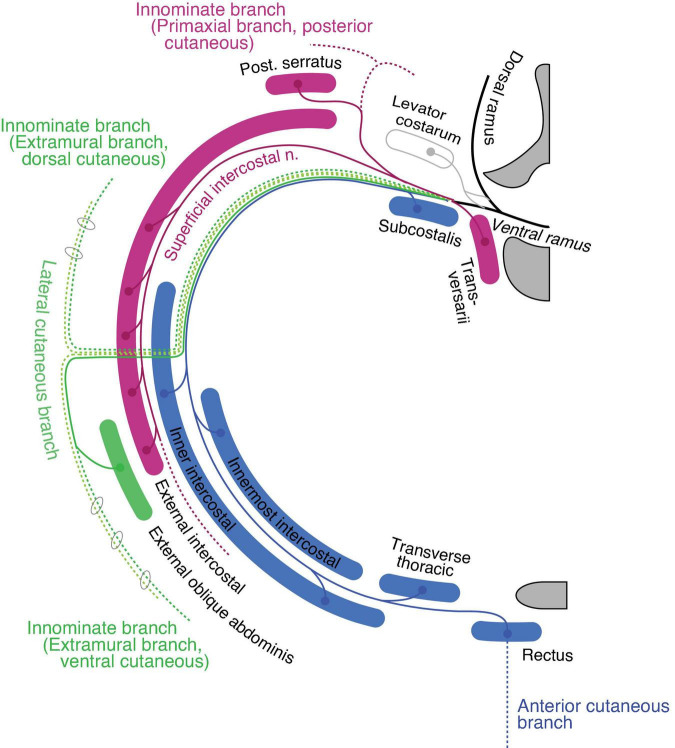
Hypothetical innervation pattern of the three nerve components of the ICN in the mid-thoracic region. The purple color represents primaxial nerve branches and muscles. The blue color represents intramural branches and muscles within the body wall. The dark and light green lines are for the extramural and LCB, fasciculated in the thoracic region. The dotted lines are cutaneous branches. The vertebra (upper right) and sternum (lower right) are dark gray in color. Because the muscles and nerves are projected onto a single transverse plane (around Th5–6), not all of them are necessarily localized to the same transverse section as illustrated.

The ventral rami radiate first-order branches to the anterior set of the intertransversarii muscle in the deep costal class, the serratus posterior in the external oblique class, and the primaxial anterior branch as well as the superficial ICN to innervate the external intercostal muscle (Figure 9). The primaxial anterior branch runs first in the deep intermuscular space between the innermost and inner intercostal muscles. It then relocates to the superficial intermuscular space between the inner and external intercostal muscles. It ends as distinctive anterior cutaneous branches from the canonical ones in the upper thoracic region.

The inner and innermost intercostal muscles are abaxial, and belong to the internal oblique and transversus classes. They are innervated by the canonical ICN (the intramural branch; Figure 9). The subcostalis and transversus thoracis muscles are on the same transverse plane as the innermost intercostal muscle; hence, they belong to the transversus class and are innervated by the canonical ICN.

The external oblique abdominis muscle belongs to the extramural muscle class, and receives a motor branch from the LCB in the lower thoracic region (Sato, 1973; Schlenz et al., 1999; Figure 9). There are no ventral extramural class muscles in the lower thoracic region. In the upper thoracic region, the pectoral major and minor muscles belong to the limb flexor and ventral extramural classes, respectively. The rationale for this allocation of two pectoral muscles lies in the innervation patterns of the two muscles. The pectoralis major receives the innervation of the pectoral nerve from the posterior surface of the muscle belly, whereas the pectoral minor is innervated by the pectoral nerve from the anterior surface. The parallel nature of the building unit in our model indicates that the limb muscle class is innervated from the posterior surface, and the extramural muscle class receives innervation from the anterior surface of the muscle (Figure 7). This rationale is also applied to the external oblique abdominis muscle, which belongs to the extramural muscle class; the external oblique abdominis receives innervation from the anterior surface via the LCB.

The general perception of the cutaneous sensory innervation on the thoracic wall is that the LCB innervates the lateral portion and the anterior cutaneous branch innervates the anterior portion. However, our model hypothesizes that three more cutaneous branches exist on the thoracic wall; the dorsal and ventral extramural cutaneous branches, and the primaxial anterior branch. Some cutaneous branches with supposed innervation patterns have been reported (Aizawa and Kumaki, 1996; Figure 9). Further study is necessary to confirm whether the suspected cutaneous branches are present or not.

#### Lumbar region

**FIGURE 10:**
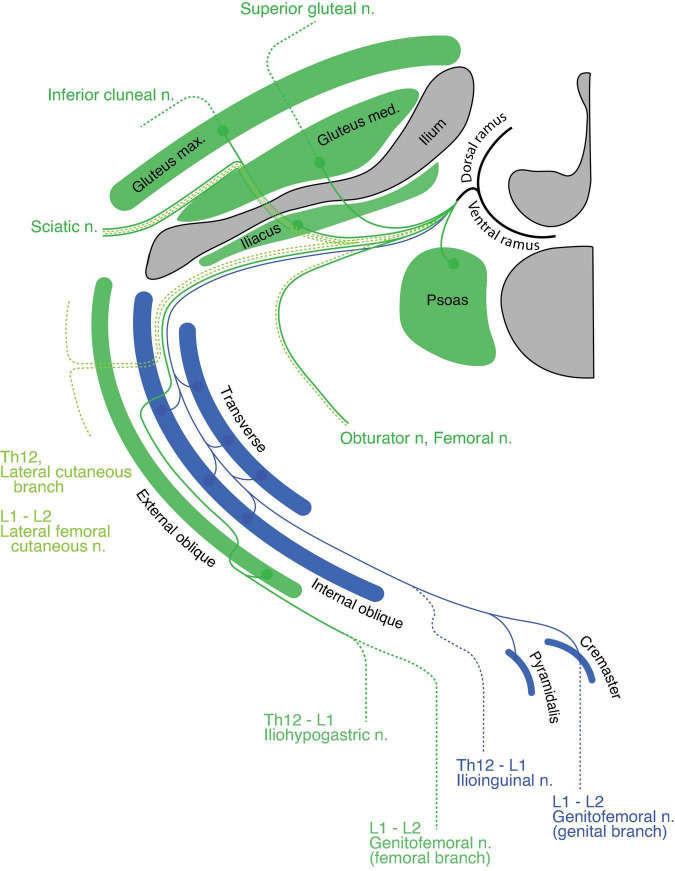
Hypothetical innervation pattern in the upper lumbar region. Nerve branches are super-imposed on a hypothetical single plane; therefore, not all of them necessarily exist on the same transverse plane. The named nerve branches are as indicated. The blue color represents the intramural branch and its target muscles. The dark green color is for the extramural branch and its target muscles. The light green color represents the LCB. The dotted segments of the lines are cutaneous branches. The bony structures are shaded in dark gray.

In the upper lumbar region, the quadratus lumborum is the only primaxial muscle, which belongs to the dorsal subgroup (Figure 10). The gluteus medius and minimus muscles are in the dorsal extramural class because they are innervated from the anterior surface by the superior gluteal nerve, whereas the tensor fascia lata belongs to the dorsal limb extensor muscle class because of the innervation from the posterior surface by the superior gluteal nerve (Figure 10). The gluteus maximus, which is innervated by the inferior gluteal nerve, belongs to the muscle class of the dorsal limb extensor (Figure 10). Regarding the ventral subgroup, the iliacus and psoas belong to the extramural and limb flexor muscle classes, respectively, because of the innervation by the terminal branches from the lumbar plexus to the anterior surface of the iliacus and the posterior surface of the psoas muscles (Figure 10).

The spinal nerve from the lumbar spinal segment 1 (L1) usually bifurcates into the iliohypogastric and ilioinguinal nerves (Standring, 2015). The iliohypogastric nerve is the extramural branch in our model (Figure 10). It conveys motor innervation to the external oblique abdominis muscle and sensory fiber to the anterolateral portion of the lower abdomen. Contrary to the general description in standard textbooks, the iliohypogastric nerve itself does not contain motor fibers to the internal oblique abdominis or transverse muscles, because these two muscles belong to different classes: the internal oblique and transverse classes, respectively. On the other hand, the ilioinguinal nerve is homologous to the intramural branch, and conveys motor innervation to the internal oblique and transverse abdominis muscles (Figure 10). The ilioinguinal nerve then ends as a sensory nerve to the skin around the genital area of the abaxial domain. We reasoned that merging the peripheral branches from the ilioinguinal nerve with those of the iliohypogastric nerve creates the appearance that the iliohypogastric nerve innervates the two deep abdominal muscles. In the lower abdomen, just above the pubic symphysis, the layered organization of the rectus muscles can be observed; the pyramidalis muscle sits on the anterior surface of the rectus abdominis. The innervation to the pyramidalis muscle differs among reports and textbooks; it is innervated by either the subcostal nerve or the ilioinguinal and genital branches of the genitofemoral nerves (Tokita, 2006: Standring, 2015). Because all of these nerves are intramural branches in our model, the pyramidalis muscle belongs to the superficial muscle of the parallel pair of the deep rectus muscle class.

Standard textbooks describe the genitofemoral nerve as being the main trunk from the L2 axial level, which bifurcates into the genital and femoral branches. In our model, the genital branch is a homolog to the intramural branch, and the femoral branch is a homolog of the extramural branch (Figure 10). The separation of the genitofemoral nerve into different nerve branches reflects said branches’ trajectories within the abdominal wall. The motor branch of the genital branch runs through the inguinal canal to innervate the cremaster muscle (a derivative of the internal oblique muscle), and the sensory branch exits out of the superficial inguinal ring to innervate the skin around the external genitalia. On the other hand, the femoral branch, which has no association with the abdominal muscles, runs on the fascial surface of the psoas major toward the upper medial thigh behind the inguinal ligament (the aponeurosis of the external oblique muscle).

The muscles in the anterior thigh (dorsal limb muscle subclass) are innervated by the extramural branches (the femoral and obturator nerves) that run along the LCBs from the third and fourth lumbar spinal nerves (Figure 10). The dorsal and ventral sensory extensions of the extramural branches are the posterior and lateral femoral cutaneous nerves, respectively (Figure 10). This assignment of cutaneous branches is reasoned by the observation that the posterior femoral cutaneous nerve sometimes originates in the inferior gluteal nerve, which is the extramural branch (Akita et al., 1992).

#### Pelvic region

**FIGURE 11:**
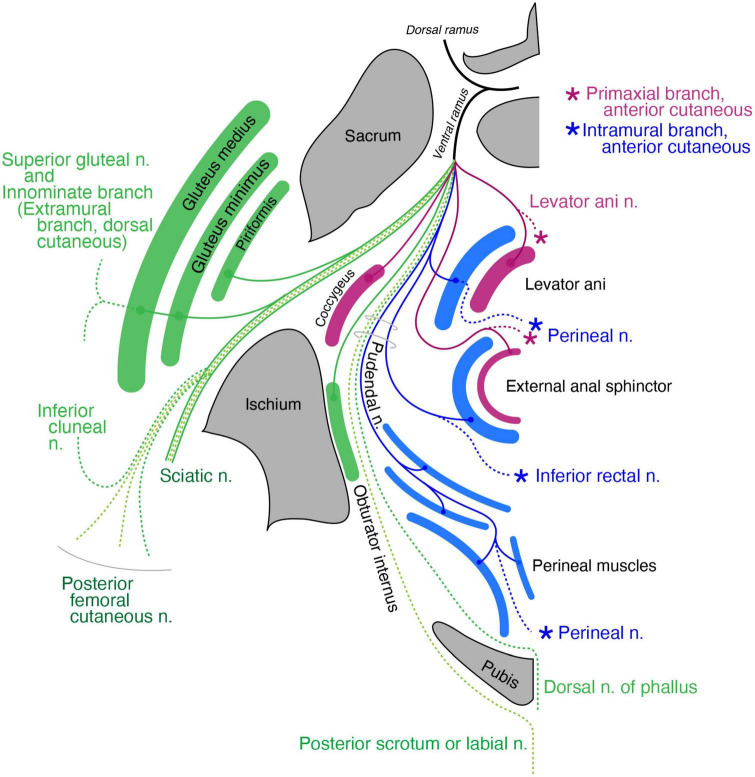
Schematic illustration of the innervation pattern in the pelvic region. The superior gluteal nerve (dark green branch) innervates the gluteus medius and minimus, and its cutaneous extension is innominate (dashed lines). The levator ani muscle, including the primaxial (purple) and abaxial (blue) parts, are differentially innervated by the primaxial (purple) and abaxial (blue) anterior branches, respectively. Similarly, the subcutaneous part of the external anal sphincter (purple) is innervated by the anterior primaxial branch (purple). The anterior abaxial branch (blue, the inferior rectal nerve) innervates the rest of the external anal sphincter muscle (blue). The muscles associated with the perineal membrane are innervated by the pudendal nerve (perineal nerve, blue). The sciatic nerve is the extramural branch (dark green). The inferior cluneal nerve corresponds to the dorsal extramural cutaneous branch (dark green). The dorsal nerve of the phallus is a homolog to the ventral extramural cutaneous nerve (dark green). The posterior femoral cutaneous and posterior scrotal/labial nerves are equivalent to the LCB (light green).

The coccygeus muscle is the only primaxial muscle in the pelvic wall (Figure 11). Regarding the muscles in the extramural class in the pelvic wall, the dorsal subgroup includes the gluteus medius, minimus and piriformis, all of which are innervated by the superior gluteal nerve or terminal branches from the sacral plexus (Figure 11). The superior gluteal nerve has a terminal sensory extension (Akita et al., 1992). The ventral subgroup of the extramural class includes the inferior and superior gemellus. The limb flexor class includes the quadratus femoris, obturator internus, and obturator externus (Figure 11).

Regarding the muscles in the pelvic floor, two different innervation patterns exist: innervation by the pudendal nerve and direct branches from the sacral plexus (Figure 11). In Gray’s Anatomy (Standring, 2015), the main nerve branch to the levator ani muscle diverges from the pudendal nerve, and is called the inferior rectal nerve. In addition, the levator ani muscle is innervated by a variant branch of the inferior rectal nerve, the levator ani nerve, which diverges directly from the sacral plexus (Bustami, 1988; Schraffordt et al., 2004; Wallner et al., 2006, 2008; Grigorescu et al., 2008; Nyangoh Timoh et al., 2017). Bustami (1988) also reported innervation of the direct sacral branch to a specific part of the levator ani muscle.

Dual innervation to the external anal sphincter is also well documented; direct sacral branches predominantly innervate the subcutaneous part of the external anal sphincter, and the branches from the pudendal nerve innervate the remaining portion of the external anal sphincter (Schraffordt et al., 2004; Wallner et al., 2006, 2008; Plochocki et al., 2016).

Our model postulates two things: (1) the rectus abdominis muscle consists of a superficial primaxial part and a deep abaxial part and (2) the primaxial and abaxial rectus muscles are differentially innervated by respective anterior branches (Figure 7). Accordingly, the subcutaneous part of the external anal sphincter and a specific portion of the levator ani muscle, both of which are innervated by the direct branches from the sacral plexus, correspond to the primaxial rectus muscle (Figure 11). The other portions of the external anal sphincter and the levator ani muscles that are innervated by the pudendal nerve (the inferior rectal and perineal nerves) correspond to the abaxial rectus muscle (Figure 11). Thus, our model may explain the biological significance of the dual innervation patterns in the pelvic floor. On the other hand, the perineal muscles belong exclusively to the abaxial domain (Valasek et al., 2005). Therefore, our model also explains the solo innervation to the perineal muscles by the perineal nerve from the pudendal nerve, which is the intramural branch, an ICN homolog (Figure 11).

The terminal branches of the pudendal nerve are the dorsal nerve of the phallus and the perineal nerve after the divergence of the inferior rectal nerve (Standring, 2015; Plochocki et al., 2016). The perineal nerve travels anteriorly, passing between the superficial and deep transverse perineal muscles, runs superficially to the surface of the perineal membrane, and terminates as the posterior scrotal (labial) nerve. Because this is similar to the ICN, which runs between the inner and innermost intercostal muscles, we allocated the superficial transverse perineal and cavernous perineal muscles to the internal oblique muscle class and the deep transverse perineal muscle to the transverse muscle class (Figure 11). The perineal nerve, which runs along the terminal end of the pudendal artery, mirrors the ICN that runs along the intercostal artery. Thus, the perineal nerve is the intramural branch in our model, and an intercostal homolog in the pelvic region.

The dorsal nerve of the phallus runs deep and anteriorly through the urogenital diaphragm to the glans, and we allocated the dorsal nerve of the phallus to the ventral extramural cutaneous branch (Figure 11). The dorsal extramural cutaneous branch in the pelvic region corresponds to the inferior cluneal nerve (Figure 11). The posterior femoral cutaneous nerve corresponds to the dorsal division of the LCB (Figure 11). Finally, the posterior scrotal or labial nerve corresponds to the ventral division of the LCB of the spinal nerve (Figure 11). The rationale for distributing the cutaneous branches is as follows. The dorsal nerve of the penis runs on the corpus cavernosum under the Buck’s fascia (in the deep fascial compartment). In contrast, the posterior scrotal or labial nerve is inside the subcutaneous tissue (outside the Buck’s fascia). These trajectories fit our criteria for the lateral cutaneous and extramural cutaneous branches.

The subdivisions of the external anal sphincter and levator ani muscles correspond to the respective rectus abdominis muscle in the primaxial and abaxial domains, according to innervation by the pudendal nerve or direct branch from the sacral plexus (Figure 11 and Supplementary Tables 1, 2).

The body wall organization of the perineum is unique; the anterior portion of the perineum has an additional body wall (the urogenital diaphragm) to the wall of the levator ani muscle (Standring, 2015). Our model illustrates a unique innervation pattern in the pelvic floor; the anterior primaxial branch is direct and independent of the abaxial anterior branch, contrary to the fasciculation of the two anterior branches in other axial levels. In more detail, the pudendal nerve (abaxial anterior branch) exclusively runs to the abaxial muscles by releasing the primaxial anterior branches to the superficial rectus muscle derivatives at the root segment of the sacral spinal nerves. Given the connection between the external anal sphincter and perineal muscles, the solo innervation to the perineal muscles by the perineal nerve suggests that the urogenital diaphragm may be the anterior expansion of the abaxial body portion in the pelvic floor due to cloacal separation.

#### Cervical region

In the cervical and brachial regions, from C3 to C8, the scalene muscles, longus capitis, and longus colli are innervated by non-plexus forming branches from the root of the spinal cord. They thus comprise the primaxial muscle group in our model. The deep prevertebral fascia enwraps these and the intrinsic back muscles around the vertebrae, and may demarcate the primaxial domain in the cervical region (Lindner, 1986; Guidera et al., 2014; Kitamura, 2018).

**FIGURE 12:**
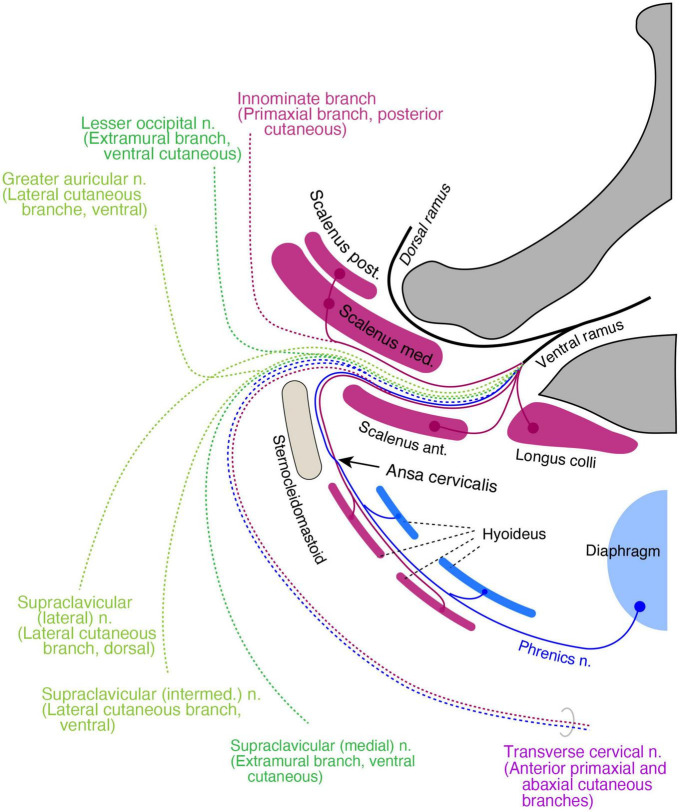
Hypothetical distribution of three nerve components in the cervical region. The positions of all branches and muscles are projected to a transverse plane of the mid neck. The primaxial motor branches (purple) include direct twigs to the scalene, longus colli, and anterior primaxial branch to the superficial infrahyoid muscles (purple). The intramural motor branches (blue) innervate deep infrahyoid muscles and the diaphragm (blue). The cutaneous branches are dotted lines and color-coded: purple for primaxial branches, blue for the abaxial anterior branch, dark green for the extramural branches, and light green for the LCBs. The posterior primaxial cutaneous branch (purple dotted line) is innominate. The transverse cervical nerve is a combination of the primaxial and abaxial anterior branches.

The rectus muscle derivatives in the neck are also in a two-layered structure, similar to the pelvic region. The superficial long rectus muscles bridge over the thyroid cartilage from the sternum or scapula to the hyoid bone (sternohyoid and omohyoid), and the deep short rectus muscles connect the thyroid cartilage to the hyoid bone (thyrohyoid) or the sternum (sternothyroid). We allocated the surface rectus muscles as primaxial (superficial rectus class) and the deep muscles as abaxial (deep rectus class; Supplementary Table 1). Because the diaphragm is considered a deep rectus muscle, the phrenic nerve is equivalent to the canonical ICN homolog (Figure 12).

The muscular organization of the neck is significantly different from that of the other body regions; the neck mostly lacks a muscular wall. Similar to the pelvic floor, the primaxial anterior branch is also free from the abaxial anterior branch in the cervical region, presumably because there are no constraints requiring two nerve bundles to be fasciculated together due to the lack of a muscular wall. However, even though there is no muscular wall in the neck, the primaxial anterior branch might still change the trajectory from deep to superficial intermuscular spaces that conceptually exist in the neck wall, resulting in an intersection with the abaxial anterior branch, the ansa cervicalis.

It is challenging to allocate the named cutaneous branches to the three nerve components in our model, because there are no intermuscular spaces as indicators. Furthermore, the sensory branches from the upper cervical spinal cord are split into ventrally directed (the transverse cervical and supraclavicular nerves) and dorsally directed (the lesser occipital and greater auricular nerves) groups; nevertheless, all the branches diverge from the ventral aspect of the spinal roots. This bisection makes allocation more difficult.

For the purpose of branch distribution, we postulate that cervical cutaneous nerves are composites. The transverse cervical, lesser occipital, and greater auricular nerves often duplicate or triplicate (Gupta et al., 2013; Ravindra et al., 2014). This numerosity suggests that the cervical cutaneous nerves may contain multiple nerve elements that may not necessarily be fasciculated under certain conditions. With this postulation, we feel that it is appropriate to apply our model to the gross anatomy of the branching pattern in the neck region, based on the ramification and distribution patterns of the cutaneous nerve.

The transverse cervical nerve is a ventrally oriented pair of anterior primaxial and abaxial branches (Figure 12). The lesser occipital and medial supraclavicular nerves are the dorsal and ventral components of the extramural cutaneous branches (Figure 12). The greater auricular and the intermediate branches of the supraclavicular nerves are the bisected ventral subdivisions of the LCB (Figure 12). The lateral supraclavicular nerve is the dorsal subdivision of the LCB (Figure 12). Our model suggests that the innominate primaxial cutaneous branch is in the posterior cervical triangle of the neck, although some of these nerve branches have already been identified in other primates (Figure 12; Kato et al., 1990).

#### Brachial region

**FIGURE 13:**
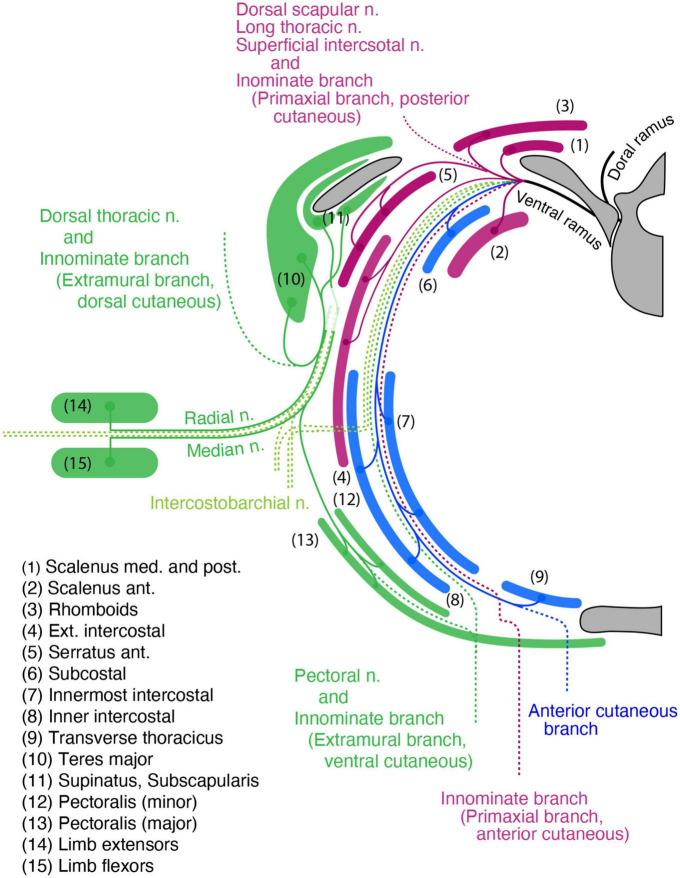
The hypothetical branching pattern of the brachial spinal nerves projected onto a transverse plane around the level of the fifth thoracic vertebra. The primaxial, intramural, and extramural branches, as well as their target muscles, are color-coded: purple for primaxial, blue for intramural, and dark green for extramural components. The dotted lines are the cutaneous branches. The names of the muscles are numbered. Primaxial branches: direct branches to the scalenus muscles, the long thoracic nerve to the serratus anterior muscle, and the dorsal scapular nerve to the rhomboids. The posterior primaxial cutaneous branch is innominate. The extramural branches are the thoracodorsal nerve to the latissimus dorsi and teres major, subscapular nerve to the subscapularis, and pectoral nerves to the pectoralis. The LCB carries extramural motor innervations into the radial and median nerves in the upper limb. The cutaneous extensions of the extramural branches are innominate.

The levator scapular, rhomboid, and anterior serratus muscles are primaxial, and belong to the external oblique class (Figure 13). The segmental branches to these three muscles do not maintain individuality. They are united as the dorsal scapula or long thoracic nerves, which diverge from the point just proximal to the cord segment of the brachial plexus. The union of the branches highlights significant differences in these two nerves from the other segmental branches. The true segmental branches innervate the scalene muscles (anterior scalenus in the longus-transversus class, and middle and posterior scaleni in the external oblique class; Figure 13).

The extramural branches innervate the ventral subgroup, including the pectoralis minor and subclavius, as well as the dorsal subgroup, including the teres minor (Figure 13). The limb flexor muscle class includes the spinatus and pectoralis major (Figure 13). The limb extensor muscle class includes the latissimus dorsi, teres major, subscapularis, and the deltoid (Figure 13). The extramural branch also innervates all upper limb muscles (Figure 13). The sensory termination of the pectoral nerve (ventral extramural) has already been reported (Koizumi and Horiguchi, 1992). The nerve branches piercing the rhomboid muscle from the ventral ramus, which are distributed throughout the lateral portion of the back, may correspond to the sensory termination of the dorsal extramural branch (Aizawa and Kumaki, 1996).

Because no muscle or dermis is associated with the ventral body wall of the brachial region, there are no branches equivalent to the canonical ICN from the brachial plexus in our model (Figure 13).

## Discussion

### Development of the intercostal nerve

The studies of the spinal nerves have primarily focused on the plexus-forming regions. The paucity of developmental studies on the ICNs might reflect their very simple morphology. However, Nakao and Ishizawa (1994) reported that developing ventral rami of the mouse intercostal nerves diverged into the three primary branches, including the branch to the external intercostal muscle, lateral cutaneous branch, and anterior branch of the intercostal nerve. The differences between their study and ours are that they did not confirm the dual innervation of anterior branches of the intercostal nerves to the anterior midline from E13 embryos. Because they used the acetylcholine esterase-histochemical method to visualize the neuronal elements, it was challenging to demonstrate the entire innervation patterns of the intercostal nerve. However, we could reanalyze the intercostal nerve development with the recent development of whole tissue-clearing methods. Our deep observations using transparent specimens, together with the retrograde labeling analysis, revealed a previously unknown innervation pattern of two different branches of the ICN from the MMCm and MMCl to the ventral midline.

We hypothesize that Lhx3-positive MNs innervate the primaxial muscle in our model, and also showed that the superficial ventral branch is from Lhx3-positive MNs in the mouse embryo. Accordingly, the innervation of the ventral midline region by the superficial anterior branches inevitably introduces a hypothetical enclave of the primaxial domain in the ventral midline region. However, the ventral midline region is believed to be entirely abaxial, and no such muscle exists in the abdominal region (Durland et al., 2008). Our observations also revealed retraction of the superficial ventral branch in the mouse embryo. Programmed cell death occurs among spinal motor neurons of mouse embryos from E13 to E15 (Lance-Jones, 1982; Yamamoto and Henderson, 1999). The time-coincident events between the neuronal origins and processes indicate that the loss of the superficial branch may result from the death of the parental motor neurons, plausibly because of the absence of primaxial target muscles in the ventral midline region. Likewise, in the thoracic region of the human body, the superficial anterior (primaxial) branch may degenerate because of the absence of the primaxial muscles in the anterior body region, resulting in the widely perceived pattern of the single anterior branch of the ICN.

### A proposed simple schema for the columnar organization of spinal motor neurons

Medial motor column is divided into the medial and lateral portions based on the relative positions of the motor pools. The medial portion of the MMC (MMCm) contains MNs that send axons through the dorsal rami to the epaxial muscles, whereas the lateral portion of the MMC (MMCl) contains the MNs innervate the hypaxial muscle in the body wall via the ventral root (Callister et al., 1987; Gutman et al., 1993; Kitamura and Richmond, 1994; Vanderhorst and Holstege, 1997; Watson et al., 2009).

However, recent molecular studies revealed that the traditional term, the MMC, does not adequately reflect the development context; the MNs that projected the axons to the hypaxial body wall were more closely associated with limb muscle-innervating MNs than those for epaxial muscles (Dasen et al., 2008; Rousso et al., 2008). Therefore, a new term, the hypaxial motor column, has been proposed to highlight the MNs that innervate the body wall muscles in the MMCl from those that innervate the epaxial muscle in the MMCm in the thoracic region (Dasen et al., 2008; Agalliu et al., 2009).

Using traditional epaxial/hypaxial muscle distinction has also caused confusions in characterizing target muscle types of MNs in respective motor columns of the spinal cord. As described previously, the ventral rami contain the innervation from the MMCm (Stillhard, 1981; Tsuchida et al., 1994; Luxenhofer et al., 2014; Nagashima et al., 2020). Nagashima et al. (2020) proposed that the shoulder muscles that are innervated by MMCm MNs are distinguished as the transitional type of muscles between epaxial and hypaxial.

To resolve the trouble of using traditional terminology, we focused on the embryonic origins of the bones at the muscle insertion points. Based on the primaxial or abaxial origins of bones, nine types of connectivity exist within the trunk, or between the trunk, and girdle and limb (Table 1). As shown in Table 1, the expression of either the LMC marker Foxp1 or the MMCm marker Lhx3 in MNs is highly correlated with the embryonic origins of the bones to which the target muscles are inserted; the MNs that innervate the muscles that are inserted into the primaxial bone express Lhx3 and MNs that innervate abaxial bone-connected muscles are FoxP1-positive. An exception is the intercostal muscles; they belong to both domains, although the ribs are entirely primaxial (Durland et al., 2008). The MNs that innervate the intercostal muscles are located in the MMCl, and are Lhx3- and Foxp1-negative (Table 1).

**TABLE 1:** Marker expression patterns in MNs innervating muscles spanning between various bones in the trunk and limb.

Connectivity	Muscle example	Lhx3 or Foxp1 expressions in MNs	Our observation and reference
Vertebrae (primaxial) to vertebrae (primaxial)	Longus colli	Lhx3	Figure 6A; Nagashima et al., 2020
Vertebrae (primaxial) to ribs (primaxial)	Scalenus	Lhx3	Figure 6B
Vertebrae (primaxial) to girdle (primaxial)	Rhomboid	Lhx3	Tsuchida et al., 1994; Nagashima et al., 2020
	Levator scapulae		Nagashima et al., 2020
	Quadratus lumborum^a^		Figure 6C
Ribs (primaxial) to girdle (primaxial)	Anterior serratus	Lhx3	Figure 6D; Nagashima et al., 2020
Vertebrae (primaxial) to limb (abaxial)	Latissimus dorsi^b^	Foxp1	Nagashima et al., 2020
	Psoas		Figure 6E
Rib (primaxial) to girdle (abaxial)	Subclavius	Foxp1	Figure 6F
Ribs (primaxial) to limb (abaxial)	Pectoralis major	Foxp1	Figure 6G; Nagashima et al., 2020
Girdle (abaxial) to limb (abaxial)	Supraspinatus	Foxp1	Figure 6H
Ribs (primaxial) to ribs (primaxial)	Intercostal muscles^c^	Double-negative^d^	Figure 3C

^a^The expression of Prx1 (a marker for abaxial connective tissue) is diffuse at the insertion point of the quadratus lumborum (Durland et al., 2008).

^b^Primaxial at the origin and distally abaxial in the latissimus dorsi (Durland et al., 2008).

^c^Proximal primaxial and distal abaxial portions in the intercostal muscles (Durland et al., 2008).

^d^The motor origin of the superficial ICN is Lhx3-positive.

Therefore, we put forward a more straightforward characterization of target muscle varieties for the three principal motor columns of the spinal cord. First, segmental branches from the Lhx3-positive MNs in the MMCm innervate the primaxial muscles inserted into the primaxial (portion of) bones. Second, MMCl MNs, both Lhx3- and Foxp1-negative MNs, send intramural branches to the muscles in the abaxial domain within the body wall. Lastly, the Lhx3-negative and Foxp1-positive MNs in the LMC send extramural branches to the abaxial muscles outside the body wall (the girdle and limb). Accordingly, the three principal columns correspond to the neuronal sources of the three nerve components in our hypothetical model. Our characterization of the motor column follows the plausible scenario of an evolution of the three motor columns (Dasen and Jessell, 2009).

Lastly, it should be noted that the MNs that innervate rhomboid and serratus anterior muscles are not in the MMCm but the lateral side of the ventral horn (LMC), although they are Lhx3-positive (Tsuchida et al., 1994; Nagashima et al., 2020; and our observation in Figure 6D). Because of the localization of the innervating MNs in the outlier, these two muscles may be intermediate muscles between the primaxial and abaxial muscles. The morphology of the innervating branches to the rhomboid and serratus anterior muscle reflects the intermediate nature of these muscles. The innervating branches of the two muscles arise segmentally from the brachial spinal nerves but merge into the dorsal scapular and long thoracic nerves.

### Sensory innervation pattern

The nature of the building unit in our model presumes eight essential cutaneous innervations from the ventral rami all along the body axis (Figure 7). From dorsal to ventral ramification, the eight cutaneous branches are:

1. The dorsal segmental branch for the primaxial domain.
2. The cutaneous branch from the dorsal extramural branch.
3. The dorsal subbranch of the lateral cutaneous branch.
4. The ventral subbranch of the lateral cutaneous branch.
5. The cutaneous branch from the ventral extramural subbranch.
6. The anterior cutaneous branch of the canonical intercostal nerve.
7. The anterior cutaneous branch from the primaxial anterior branch.
8. The ventral segmental branch for the primaxial domain.

The number of cutaneous branches in our model is far higher than the three currently known branches in the thoracic region (the dorsal and ventral subbranches of the lateral cutaneous branch and the anterior cutaneous branch of the intercostal nerve). Further study is necessary to confirm whether our hypothesized cutaneous branches are present or not. However, some cutaneous branches with suspected innervation patterns have been reported (Aizawa and Kumaki, 1996).

### An alternative view on the muscular organization of the body wall

**FIGURE 14:**
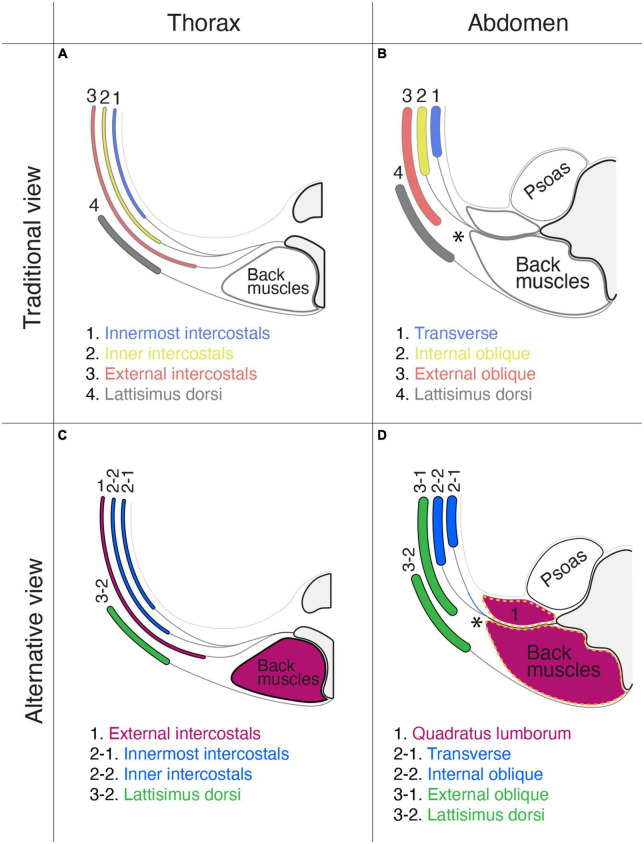
Comparison of the layer organization of the body wall muscles between the traditional view and our view. The muscle homology between the thorax **(A,C)** and abdominal wall **(B,D)**. The homologous muscles are color-coded. In the traditional view **(A,B)**: (1) the innermost intercostal muscle corresponds to the transverse abdominis muscle; (2) the internal intercostal muscle corresponds to the internal oblique abdominis muscle; (3) the external intercostal muscle corresponds to the external oblique abdominis muscle; and (4) the latissimus dorsi is outside the body wall. In the proposed alternative view **(C,D)**: (1) the external intercostal muscles are equivalent to the quadratus lumborum; (2) the internal and innermost intercostal muscles correspond to the internal oblique and transverse abdominis; and (3) the external oblique abdominis is closely related to the latissimus dorsi. In panel **(D)**, the thoracolumbar fascia is indicated by broken orange lines. In panels **(B,D)**, asterisks indicate the lateral raphe.

The thoracic, abdominal, and pelvic wall muscles have three layers (Nishi, 1938; Hall et al., 2017). The traditional view of the homology of muscles between the thoracic and abdominal walls is that the external intercostal muscles correspond to the external oblique abdominis, the inner intercostal muscles to the internal oblique abdominis, and the innermost intercostal muscles to the transverse abdominis (Figures 14A, C). This perceived conception is based on the topology of muscles in the body wall and partly comes from the running orientation of muscle fibers. However, our view is that the external intercostal muscles are homologous to the quadratus lumborum (innervation by segmental branches to muscles in the primaxial domain), the inner intercostal muscles are homologous to the internal oblique abdominis, and the innermost intercostal muscles are homologus to the transverse abdominis (innervation by the intramural branch). The external oblique abdominis is not homologous to the external intercostal muscles; instead, it is closely related to girdle muscles such as the latissimus dorsi (innervation by the extramural branch; Figures 14B, D). Thus, muscle classification based on the developmental origins and corresponding innervations by the three components illustrates a novel configuration of body wall musculatures. The external intercostal muscles (the primaxial muscle) are sandwiched between the latissimus dorsi, and inner and innermost intercostal muscles (the abaxial muscles) at the thoracic level. At the lumbar level, the primaxial muscles (the intrinsic back and quadratus lumborum muscles) are adjoined with the abaxial muscles (the transverse and internal oblique muscles), creating the lateral raphe.

We want to emphasize that the thoracolumbar fascia may wrap the muscles in the primaxial domain at the lumbar level. The thoracolumbar fascia invests the intrinsic back muscles and quadratus lumborum, and the external oblique abdominis muscle is located entirely external to the thoracolumbar fascia (Figure 14D; Willard et al., 2012; Standring, 2015).

Hall et al. (2017) previously documented a homology of the muscular organization between the pelvic and abdominal regions in the body wall. We here provide a different view of muscle homology between the pelvic and thoracic regions, based on the innervation patterns of the different classes of nerve branches. The homologous muscles between the two segments of the body wall are as follows: (1) the innermost intercostal muscle (transverse class, abaxial) to the deep transverse perineal muscle; (2) the internal intercostal muscle (internal oblique class, abaxial) to the superficial transverse and perineal muscles; and (3) the external intercostal muscle (external oblique class, primaxial) to the coccygeus muscle.

### Evolutionary significance

Also of significance is that our model explains the evolutionary aspect of the body wall organization and cognate branching patterns. The primaxial branches innervate the muscles that constitute an essential set for respiration and locomotion. The selective innervation to the abaxial muscles, including the body wall and limb muscles by the intramural and extramural branches, is possibly associated with terrestrial adaptation. The intramural branches innervate the body wall muscles that are also involved in breathing, but through more active and efficient manners, including changing the volume of the chest cavity via movement of the diaphragm, active exhalation by the inner and innermost intercostals, and abdominal pumping by the oblique and transverse abdominis. The extramural branches innervate the girdle and limb muscles that are required for more refined apparatus for locomotion. Thus, our model will provide fundamental grounds for comparative studies on evolutionary changes in the vertebrate body.

### Reasoning for the variable branching pattern of the lumbar plexus

It is clear that there are developmental reasons for variations and anomalies in branching patterns of the spinal nerve; examples caused by mutations in individual genes, such as those reported in experimental animals, are seldomly observed in the human body (Philippidou et al., 2012). Nevertheless, we have had no foundation to explain the developmental reasoning for diverse branching patterns.

The lumbar plexus is probably the most frequently mutated peripheral nerve. By using the lumbar plexus, we explain how our model explains the reasons for the diverse branching patterns.

**FIGURE 15:**
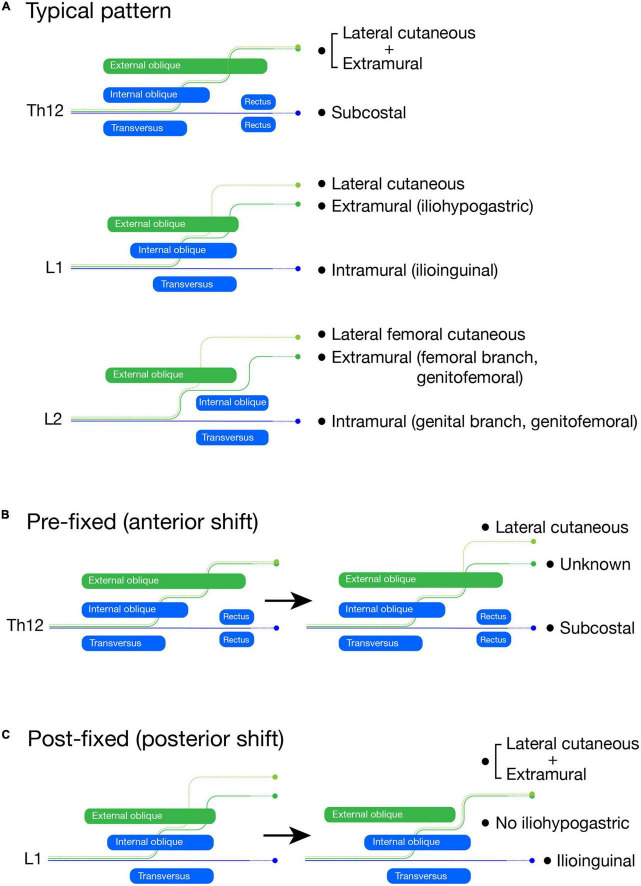
Schematic illustration of the lumbar plexus formation. The LCBs are light green, the extramural branches are green, and the intramural branches are blue. In the typical branching pattern **(A)**, the Th12 is the subcostal nerve (blue, intramural branch) with the lateral cutaneous and extramural branches, which are tied together. The L1 spinal nerve bifurcates into the iliohypogastric (extramural branch) and ilioinguinal (intramural branch) nerves. The LCB is usually present from the L1 nerve. The L2 spinal nerve also bifurcates into the femoral (extramural branch) and genital (intramural branch) branches of the genitofemoral nerve. The LCB from the L2 nerve is equivalent to the lateral femoral cutaneous nerve. In the pre-fixed plexus **(B)**, the Th12 (subcostal nerve) bifurcates into the extra and intramural branches like the L1 nerve in the typical pattern. However, we are unable to identify the extramural branch as the iliohypogastric nerve, although we can identify the intramural branch as the subcostal nerve and the LCB. In the post-fixed plexus **(C)**, the L1 spinal nerve does not bifurcate, which is similar to the subcostal nerve. The ilioinguinal nerve and LCB are there, but the iliohypogastric nerve is not; it is fasciculated with the LCB.

The lower abdomen is a transitional region from the body to the limb-girdle. However, the exact borderline between the lower abdomen and the girdle is unclear. Our model reveals that the lower girdle covers the “true” body wall within the abaxial domain at the lumbar level (Figures 10D, 14). This section describes the logic of how variable innervation patterns of the lumbar plexus emerge from the positional fluctuation of the front line between the body and lower limb-girdle (Figure 15).

The branches from the L1 and 2 spinal nerves, the iliohypogastric, ilioinguinal, and genitofemoral nerves, are highly variable in many aspects of innervation patterns (Al-dabbagh, 2002; Peschaud et al., 2006; Ndiaye et al., 2010; Cirocchi et al., 2019). It has often been documented that the two main branches of the lumbar (L) L1 and L2 spinal nerves are reciprocal in size (Apaydin and Bozkurt, 2015). However, we do not think that the reciprocity is a mutual complementarity. A previous detailed study revealed that the size-interchange of the main branches is closely related to the axial contribution (pre-fixed or post-fixed) of the spinal nerves to the lumbar plexus formation (Kumaki, 1995). There were three main findings in that study. First, the L1 and L2 spinal nerves bifurcate into superficial and deep branches, which travel respectively through the superficial and deep intermuscular spaces of the abdominal wall. Second, they the superficial branches from the L1 and L2 spinal nerves are more conspicuous under the anterior shift (pre-fixed) of the spinal nerves that join the plexus formation (e.g., Th12 contribution), in contrast to the deep branches, which are more evident under the posterior shift (post-fixed) of the plexus-forming spinal nerves (e.g., less L1 contribution. Third, the LCB is present in the L1 spinal nerve only when the deep branch is noticeable.

In more detail, the superficial branch from the L1 spinal nerve first runs through the deep intermuscular space between the transverse and internal oblique abdominis muscles, then changes to the superficial intermuscular course between the internal and external oblique abdominis muscles. It next emerges through the external oblique aponeurosis around the anterior superior iliac spine to innervate the suprapubic skin. The nerve branch with this typical superficial trajectory corresponds to both the extramural branch in our model and the iliohypogastric nerve in textbooks (Figure 15A). On the other hand, the deep branch from the L1 spinal nerve runs through the deep intermuscular space until they reach the rectus abdominis muscle, and corresponds to the intramural branch, representing the typical ilioinguinal nerve (Figure 15A). The intramural branch from the L2 is the genital branch of the genitofemoral nerve because it goes into the inguinal canal and innervates an internal oblique derivative, the cremaster muscle (Figure 15A). The femoral branch is the extramural branch, as it runs free from the body wall and goes into the thigh by passing underneath the inguinal ligament (Figure 15A).

Our theoretical ramification model postulates that the superficial extramural branch is for the innervation of the girdle region, and the deep intramural branch is for the innervation of the body proper. Accordingly, the model explains the above reciprocal dynamic as the relative proportion of the body and girdle regions in the lower abdomen; the post-fixed plexus reflects the more abdominal portion in the lower abdomen, forming the thick intramural (deep) branches, whereas the anterior shift (pre-fixed) of the plexus formation accompanies a broader portion of the lower girdle, leading to the larger extramural (superficial) branches from the lumbar plexus (Figures 15B, C).

Under the extreme anterior shift (pre-fixed type), the Th12 nerve becomes more like the L1 branching pattern; the extramural branch is separated from the LCB (Figure 15B). However, we cannot name either branch, the iliohypogastric or ilioinguinal nerves, because they are the branches from the subcostal nerve (Th12). In the extreme case of the posterior shift (post-fixed type), the L1 spinal nerve becomes more like the subcostal nerve; the deep branch can be identified as the ilioinguinal nerve, whereas the iliohypogastric nerve (extramural nerve) is missing because it is tied together with the LCB (Figure 15C). In other words, the uppermost spinal nerve with the apparent extramural (superficial) branch provides a rough approximation of the axial position of the lower girdle. The axial shift in the plexus-formation does not occur segment by segment and generates numerous intermediate situations, which makes it challenging to identify individual branches.

The peripheral distributions of the lumbar spinal nerves are critical pieces of information to avoid surgical complications after an operation for an inguinal hernia or cesarean section. Pre-existing knowledge of the axial contribution of the lumbar spinal nerves to lumbar plexus formation may help to predict the numbers and thickness of the peripheral branches.

## Materials and methods

### Mouse embryos

We purchased pregnant mice (ICR strain) from a local vendor (SLC, Kumagaishoten) and euthanized them under deep anesthesia with isoflurane vapor. The uterus was excised and immediately soaked in ice-cold phosphate-buffered saline (PBS). The mouse embryos (E10 and E15) were then taken out of the ice-cold PBS and stored in ice-cold L-15 medium (Wako Pure Chemical, Japan) until use, and immersed in cold 0.1 M phosphate-buffered 4% paraformaldehyde (4% PA in 0.1 M PB). The Animal Experimental Committee of Fukushima Medical University approved the protocol for the animal experiments in this study (Approval number 30071).

### Retrograde labeling

After the embryos were decapitated and eviscerated, the target muscles and nerves were exposed in cold PBS, and Alexa594-conjugated Dextran (Cat# D22913, Thermo Fisher Scientific, USA) was injected using a picosplitzer (IM300, Narishige, Japan). The injected embryos were kept in oxygen-bubbled DMEM (Wako Pure Chemical, Japan) for up to 5 h at 30°C. After incubation, the embryos were fixed in 4% PA in 0.1 M PB for 5 h and then immersed in 20% sucrose in PBS overnight. The appropriate segments of the spinal cord were excised from the embryo and frozen-embedded in a mixture of 20% sucrose in PBS and Tissue-Tek embedding media (1:2 ratio; Sakura, Japan). Ten to fifteen muscles were injected with the tracer, and one-to six spinal cords with successful labeling were obtained for each muscle type.

### Immunohistochemistry and image acquisition

Whole-mount neurofilament immunohistochemistry was carried out according to the iDISCO protocol (Renier et al., 2014), except for the tissue-clearing methods. The anti-neurofilament primary antibody was purchased from Biolegend, USA (Cat# 841001, 1:500 dilution). Ten sides of the thorax were processed for each E10 and E15 embryo.

For the simultaneous detection of the MN markers in the labeled MNs, cryostat sections were cut and processed for immunohistochemistry as follows. The primary antibodies used in this study were purchased from Abcam, UK; Lhx3, a marker for MMCm MNs (Cat# ab14555, 1:1,000 dilution), Pou3f1, a marker for phrenic MNs (Cat# ab221964, 1:250 dilution), and FoxP1, a marker for LMC MNs (Cat# ab16645, 1:500 dilution). For Pou3f1 and FoxP1 immunohistochemistry, the sections were treated in 0.01 M SSC buffer (pH 7.0) for 20 min at 98°C for antigen retrieval. The sections were blocked with 10% normal goat serum in PBS with 0.1% Tween 20 (PBST) for 1 h, incubated with primary antibodies overnight at 4°C, and then incubated with Alexa 488-conjugated anti-rabbit goat antibody (Cat# A-11008, Thermo Fisher Scientific, USA) at room temperature for 3 h.

Images were obtained under a confocal microscope (A1, Nikon, Japan). For 3D reconstruction from captured images, Flurorender (Wan et al., 2009) and Adobe Photoshop applications were used, and maximum intensity projection images were created.

### Tissue-clearing with TDE

The organic solvents used in the iDISCO protocol (Renier et al., 2014), such as tetrahydrofuran and dibenzyl ether, are toxic and require special care for handling. For this reason, we used 2,2’-thtiodiethanol (TDE: Cat# 166782, Sigma-Aldrich, USA) for tissue clearing and mounting media (Staudt et al., 2007). After immunohistochemistry, the samples were fixed in 4% PA, 0.1 M PB for 30 minutes at room temperature. Following a brief wash with PBST, the samples were immersed in a graded series of TDE/water mixture with a final concentration of 97%, and mounted in a hand-made chamber. We built the chamber on a slide glass by stacking square vinyl frames that were initially developed for *in situ* hybridization (Cat# AB-0578, Thermo Fisher Scientific, USA). The depth of the room was adjusted by the number of stacking layers according to the thickness of the specimens. After placing the processed tissue samples in the chamber, it was filled with TDE and sealed with a coverslip. The specimen was therefore flattened and immobilized between the slide glass and coverslip.

Other than safety, the most significant reason why we used TDE instead of organic solvents in the original iDISCO protocol was to avoid refractive index mismatching along the light path. One of the leading causes of hampering the obtaining of clear and defined images from deep inside the sizeable whole-mount specimen was the refractive index mismatch that occurred at phase boundaries in the observation setup. Because the point spread function indicates that the magnitude of image distortions is exponentially proportional to the length of the light path, the refraction at the phase boundaries was carefully eliminated from the whole light path, especially during observation of the volumetric 3D sample. Mounting a biological specimen in the TDE-filled chamber, as described above, eventually provides refractive index mismatch-free preparation of embryonic tissues for 3D observation. As our study has demonstrated, it is possible to obtain clear and bright images of deep inside specimens with a conventional confocal microscope.

## Data availability statement

The original contributions presented in this study are included in the article/Supplementary material, further inquiries can be directed to the corresponding author.

## Ethics statement

This animal study was reviewed and approved by the Animal Experimental Committee of Fukushima Medical University.

## Author contributions

SH conceived the study design, performed the research, and drafted/revised the manuscript. TS assisted with the experiments. IW provided expertise for 3D-image acquisition. KK and NS provided knowledge and discussion on human gross anatomy. HY secured funding for the research. All authors read and approved the final manuscript.
