# Supplementary material for: A three-component model of the spinal nerve ramification: Bringing together the human gross anatomy and modern Embryology
Source: Front Neurosci. 2023 Jan 16;16:1009542. doi: 10.3389/fnins.2022.1009542 (PMC9884977; doi:10.3389/fnins.2022.1009542)
Supplement: Supplementary file 1 [file Data_Sheet_1.pdf]

Supplementary Information: Muscle Classification in the Human Body

In preparation for the detailed application of our model in the peripheral innervation pattern of the human body, we classified all target muscles in the human body into seventeen categories, in accordance with Nishi's classification (Nishi, 1938; see also Sato, 1968), with modifications. Supplementary Table 1 shows the individual members of each muscle class. Some muscles (e.g., latissimus dorsi) are partitioned into the primaxial and abaxial portions (Durland et al., 2008). We postulate that such muscles receive respective innervations by segmental and plexus-forming branches, and determined their muscle classes according to the embryonic domains at the insertion points into their respective bones.

1. Back muscles and dorsal rami (Supplementary Table 1)

The submultifidus, multifidus, longissimus, and iliocostalis classes comprise the intrinsic back muscles (primaxial muscles). These four muscle classes are divided into medial (submultifidus and multifidus classes) and lateral subgroups (longissimus and iliocostalis classes). All muscles are innervated exclusively by the dorsal rami of the spinal nerve.

2. Intertransversarii muscles and intermediate rami (Supplementary Table 1)

Multiple muscle slings between the transverse processes of the vertebrae comprise the intertransversarii muscles (Standring, 2015). The muscle complex of the intertransversarii consists of the anterior and posterior sets, between which the ventral rami of the spinal nerves run. The anterior set receives innervation from the ventral rami of the spinal nerve, and belongs

to the deep costal muscle class in our model (Sato, 1968; Standring, 2015). The posterior set further differentiates into the medial and lateral slips. The lateral slip also receives innervation from the ventral rami and is more closely related to the external intercostal muscle than to the intertransversarii muscles (Sato, 1968; Standring, 2015). Accordingly, we placed the lateral slip of the posterior set in the external oblique class. The medial slip of the posterior set corresponds to the intertransversarii muscle proper (Sato, 1968; Standring, 2015).

Sato (1971 and 1974) found that the branches to the actual intertransversarii muscle segmentally originated from the nerve root between the dorsal and ventral rami, and argued that the branches should have a unique collective name; the intermediate rami. In accordance with Sato's proposal, we placed the medial slip of the posterior set in the intertransversarii muscle class in the primaxial domain. In the thoracic region, the lateral half of the medial slip of the posterior set corresponds to the levatores costarum muscle (Sato, 1971 and 1974).

3. Body wall and appendicular muscles, and ventral rami (Supplementary Table 1)

The ventral rami of the spinal nerve innervate the muscles in the remaining twelve classes. We further separated them into either dorsal or ventral subgroups, based on their relative positions to the ventral ramus of the spinal nerve. For example, the ventral subgroup includes the anterior scalene muscle in the longus-transversus class. In contrast, the middle and posterior scalene muscles in the external oblique class belong to the dorsal subgroup. Regarding the limb muscles, the dorsal subgroup includes the extensor muscles, and the ventral subgroup contains the flexor muscles.

The primaxial muscle classes consist of the supracostal and external oblique muscle classes in the dorsal subgroup, and the superficial rectus (see section 2.1), longus-transversus, and deep

costal classes in the ventral subgroup. All of these muscles are innervated by segmental branches that are diverged from the root segment of the ventral rami.

The abaxial muscle classes include the extensor and flexor muscles in the limb, as well as dorsal and ventral extramural, deep rectus, internal oblique, and transversus classes. The limb and extramural muscle classes receive motor innervation from the extramural branch. The muscles in the deep rectus, internal oblique, and transversus classes are innervated by the intramural branch, which is homologous to the ICN. These three classes of abaxial muscles belong to the ventral subgroup.

3. 1 Rectus muscle

In the lower thoracic and upper lumbar regions, we were intrigued by the relative position of the rectus muscle to the internal oblique abdominis muscle. Above the arcuate line, the posterior lamina of the internal oblique abdominis muscle is adjoined to the posterior lamina of the rectus sheath. However, below the arcuate line, the posterior lamina of the internal oblique abdominis muscle runs anterior to the rectus abdominis muscle, fusing with the anterior lamina of the rectus sheath. This laminae arrangement of the rectus sheath indicates that the rectus abdominis muscle is anterior to, or on, the same transverse plane as the internal oblique muscle above the arcuate line. However, the rectus abdominis muscle is positioned posteriorly to the internal oblique and transverse abdominis muscles, below the arcuate line.

This topological difference in the rectus muscle may present two possibilities: the rectus abdominis muscle passes through the internal oblique abdominis muscle at the arcuate line; or there are two different, the superficial and deep, rectus muscles, which are located above and below the arcuate line. We adopted the latter interpretation in our model. We allocated the superficial and deep rectus muscles to the parallel pair of the abaxial rectus muscle class.

In addition to the abaxial rectus muscle class, we postulate another class of rectus muscles, the primaxial rectus class, in our model. The reasons for this postulation are as follows. The superficial ICN, which innervates the dorsal subgroup of muscles in the primaxial domain, occasionally innervates the rectus abdominis muscle (Kodama, 1986). Because the rectus abdominis belongs to the ventral subgroup of muscles, it seems odd that it is innervated by the superficial ICN, which is supposed to innervate the dorsal subgroup of muscles. This mismatch necessitated us to introduce an additional muscle class (e.g., superficial rectus muscle class), which is innervated by the anterior primaxial branch, in the anterior midline region all along the body axis.

4. Muscles in the pelvic floor

Compared to the definite muscular organization of the pelvic wall, that of the pelvic floor is inconsistent among standard textbooks and references, which has resulted in significant confusion in terms of denoting individual muscles. This disorder may arise from the substantial divergence of the muscular organization between recent dissection studies and the standard reference. In the current section, we introduce an alternative view of the pelvic floor muscles to those of previous studies, and provide a novel conception regarding the muscular organization of the pelvic floor.

4. 1. Levator ani muscle

In Gray's Anatomy (Standring, 2015), one of the muscular complexes in the pelvic floor, the levator ani muscle, is described to consist of three muscle slings: the iliococcygeus, pubococcygeus, and puborectalis. The conception of the three parts in the levator ani muscle dates back to a study by Thompson (1899). However, later studies revealed different landscapes

on the muscular organization of the levator ani muscle. Ayoub (1979) reported that the levator ani muscle consists of anterior and posterior parts, with three layers in the anterior part. Bustami (1988) also reported a layered organization in the anterior portion of the levator ani muscle. The details of his report are: 1) the anterior portion is thick, and is further subdivided into two layers, the deep and superficial layers; 2) the anterior fibers in both layers of the anterior portion are connected to the external anal sphincter muscle; 3) the posterior fibers in the superficial layer do not correspond to any known muscles; and 4) the deep layer in the posterior portion is equivalent to the classical pubococcygeus.

A recent 3-D reconstruction study also reported that the levator ani muscle consists of anterior and posterior portions (Wu et al., 2015). The single anterior portion is bifurcated into two discrete muscle masses in the posterior portion. The two posterior portions correspond to the classical puborectalis and pubovisceral muscles. However, the pubovisceral muscle cannot be subdivided into pubococcygeal and iliococcygeal muscles, although it is bilayered, consisting of medial and lateral layers. Moreover, other studies have reported that the puborectalis part of the levator ani muscle is morphologically and functionally related to the external anal sphincter muscle (Shafik, 1975; Fröhlich et al., 1997; Fritsch et al., 2002).

The results of the above-mentioned studies indicate the following points: 1) the levator ani muscle is multi-layered; and 2) the classical puborectalis muscle may not be a part of the levator ani muscle. Thus, detailed dissection studies have provided us with a completely different conception of the levator ani muscle organization.

4. 2. External anal sphincter

The muscle organization of the external anal sphincter also possesses a similar conflict. It has long been accepted that the external anal sphincter consists of three muscle components:

superficial cutaneous, superficial, and deep components (Standring, 2015). Wu et al. (2015) also divided the external anal sphincter into two parts: the puborectal part, which includes the puborectalis, deep and superficial muscles of the external anal sphincter, and the cutaneous part. In addition, Oh and Kark (1972) added another component of the levator ani muscle, the puborectalis muscle, to the external anal sphincter complex, for the suspected connection of the deep portion of the external anal sphincter to the puborectalis muscle, taking the number of components to four. Al-Ali et al. (2009) then reduced the previously recognized multiple parts to only two parts: the superficial cutaneous and deep parts of the external anal sphincter. The superficial part of the external anal sphincter includes only superficial cutaneous muscle, and the deep part includes classical superficial and deep muscles. Nevertheless, all studies agree that the superficial cutaneous part of the external anal sphincter is an independent muscle component.

4. 3. Composition of the pelvic floor muscles

Although the number of subdivisions of the external anal sphincter varies depending on the study, the three-layered model of the external anal sphincter is useful when focusing on its connection to neighboring muscles such as the perineal and levator ani muscles. Close examinations of the perineal regions have revealed that the deep component of the external anal sphincter was connected to the puborectal part of the levator ani muscle, and that the superficial component of the external anal sphincter was continuous with the perineal muscles (Arakawa et al., 2010; Plochocki et al., 2016). Moreover, Ayoub (1979) also reported that the pelvic and middle layers of the levator ani muscle were connected to the classical deep part of the external anal sphincter, and that the perineal layer is connected to the classical superficial part of the external anal sphincter. As described above, Bustami (1988) reported that the anterior fibers in the anterior portion of the levator ani muscle are connected to the external anal sphincter muscle.

Baramee et al. (2020) and Suriyut et al. (2020) also reported the integral architecture of the pelvic floor, which consists of the levator ani, perineal, and external anal sphincter muscles.

The above dissection and 3-D reconstruction studies revealed a novel conception of the muscular organization of the pelvic floor; the muscles of which form a multi-layered structure that connects the perineum to the anorectal regions. However, the muscle wall differentiates into morphologically segmented and functionally specialized complexes, including the perineal, external anal sphincter, and levator ani muscle, possibly because the pelvic floor muscles are responsible for particular functions such as micturition, reproduction, and defecation.

4. 4. Muscular organization of the pelvic floor (Supplementary Table 2)

Questions have arisen as to why there are various descriptions about the anatomical organization of the pelvic floor, and the naming of the muscles. As described above, the external anal sphincter and levator ani muscles consist of multiple layers. However, it is unclear whether the individual layers are subdivisions of a single muscle mass or elements that comprise muscle complexes. If the latter is the case, it is challenging to compare the subdivisions of muscles classified in various studies without adequate reference categories. For this reason, our first step was to distribute the pelvic floor muscles into our muscle classes based on the stratification patterns of muscle components and continuities with neighboring muscles.

Because the anorectal canal opens in the ventral midline of the body wall, we hypothesize that the surrounding muscle, the external anal sphincter, belongs to the rectus class. In our model, all rectus muscles belong to one of the four subclasses (a pair in the primaxial rectus and a pair in the abaxial rectus). The standard three subdivisions of the external anal sphincter muscle, the subcutaneous, superficial, and deep parts, correspond to one subclass of the superficial rectus and a pair of the deep rectus classes, respectively.

We interpret the continuations in the pelvic muscles as the muscular integration of the body wall in such a way that the internal oblique abdominis muscle fascia is connected to the rectus sheath to form the complete circle of the abdominal wall. Because of the connection between the bulbospongiosus muscle and the superficial external anal sphincter, we categorize the former as a deep rectus muscle (Plochocki et al., 2016). Based on the fiber orientations, the other perineal muscles (the ischiocavernosus and transverse perineal muscles) are placed in the internal oblique and transversus classes. Most of the levator ani muscle are distributed to the internal oblique and transverse muscle classes. Regarding the subdivisions of the levator ani muscle in Bustami's study (1988), the anterior fibers in the superficial and deep layers of the anterior portion belong to the two deep rectus classes, respectively. The posterior fibers in the deep layer of the anterior portion belong to the internal oblique class. Those of the posterior portion belong to the transverse class. A small part of the levator ani muscle (posterior fibers of the superficial perineal layer in the anterior portion), which Bustami (1988) found to not correspond to any established subdivisions of the levator ani, belongs to the longus-transversus class.

5. Evidence for the two different classes of the rectus muscle derivatives in the neck (Supplementary Figure)

The layered architecture of the rectus muscle is well preserved in the neck and pelvic regions. Our model predicts that motor neurons (MNs) innervating the superficial infrahyoid muscles express Lhx3, and that those innervating the deep infrahyoid muscles express Pou3f1 (Oct6, SCIP), which is a marker for MNs innervating not only the diaphragm (rectus derivative), but also the deep rectus muscles.

We injected a retrograde tracer into a tissue mass below the hyoid cartilage and above the trachea to test the above-mentioned prediction because the individual muscles in the infrahyoid region were too small to discern. After the injection, we observed the labeling in the hypoglossal nucleus and the ventral horn. Some of the labeled MNs within the ventral horn expressed Lhx3 in the medial side of the ventral horn (MMCm) and Pou3f1 in the lateral side. The Pou3f1-positive-labeled MNs were in a cluster of cells with high Pou3f1 expression. This expression level indicates that Pou3f1-positive MNs are in the MMCl because the expression level is low in the MMCm, but high in the MMCl (Rousso et al., 2008). The expression patterns of Lhx3 and Pou3f1 in the cervical spinal cord thus support our model. Rousso et al. (2008) reported that MNs with the MMCl profile (Pou3f1-positive) exist all along the axial levels. Our results indicate that Pou3f1 may be a marker for MNs innervating the deep rectus class of muscle in the abaxial domain.

Supplementary Tables

Supplementary Table 1 Classification of human muscles in the three component model.

			Class	Muscle	Nerve	Origin
Dorsal rami	Medial subgroup	1	Submultifidus	Rectus capitis post.	Nerve to	Root
				Obliquus capitis inferior	Nerve to	Root
				Rotatores	Nerve to	Root
		2	Multifidus	Multifidus	Nerve to	Root
				Semispinalis	Nerve to	Root
	Lateral subgroup	3	Longissimus	Obliquus capitis superior	Nerve to	Root
				Longissimus	Nerve to	Root
				Splenius	Nerve to	Root
		4	Iliocostalis	Iliocostalis	Nerve to	Root
Intermediate rami		5	Intertransversarii	Intertransversarii proper (medial slip, of the posterior set)	Nerve to	Root
				Thoracic intertransversarii (medial half, medial slip of the posterior set)	Nerve to	Root
				Levator costarum (lateral half, medial slip of the posterior set, thoracic intertransversarii)	Nerve to	Root
Ventral rami	Dorsal subgroup	6	Supracostal	Serratus posterior superior	Superfical intercostal	Root
				Serratus posterior inferior	Superfical intercostal	Root
		7	External Oblique	Cervical intertranversarii (lateral slip, posterior set)	Nerve to	Root
				Scalenus medius	Nerve to	Root
				Scalenus posterior	Nerve to	Root
				Levator scapulae	Dorsal scapula	Root
				Rhomboid	Dorsal scapula	Root
				Serratus anterior	Long thoracic	Root
				Intercostales externi	Superficial intercostal	Root
				Lumbar intertransversarii (lateral slip, posterior set)	Nerve to	Root
				Quadratus lumborum	Nerve to	Root
				Coccygeal	Nerves to	Root
		8	Dorsal extramural	Teres minor	Axillary	Cord
				Gluteus medius	Superior gluteal	Division
				Gluteus minimus	Superior gluteal	Division
				Piriformis	Nerves to	Division
		9	Limb extensor	Deltoid	Axillary	Cord
				Latissimus dorsi	Thoracodorsal	Cord
				Subscapularis	Subscapularis	Cord
				Teres major	Subscapularis	Cord
				Gluteus maximus	Inferior gluteal	DIvision
				Tensor fascia lata	Superior gluteal	Division
				Extensor muscles in the limb	Plexus	Terminal branch
	Ventral subgroup	10	Limb flexor	Supinatus	Suprascapular	Trunk
				Pectoralis major	Pectoral	Cord
				Posas	Nerve to	Division
				Quadratus femoris	Nerve to	Division
				Obturator internus	Nerve to	Division
				Obturator externus	Obturator	Branch
				Flexor muslces in the limb	Plexus	Terminal branch
		11	Ventral extramural	Subclavius	Subclavius	Trunk
				Pectoralis minor	Pectoral	Cord
				Abdominal external oblique	Intercostal (lateral cutaneous branch)	Cord
				Iliacus	Nerve to	Division
				Gemellus (inferior)	Nerve to	Division
				Gemellus (superior)	Nerve to	Division
		12	Rectus (superficial)	Infrahyoid (superficial)	Ansa cervicalis	Root
				Rectus abdominus (superficial)	Intercostal	Root
				Sphinctor ani externus (subcutaneous portion)	Nerve to	Root
		13	Rectus (deep)	Infrahyoid (deep)	Ansa cervicalis	Branch
				Rectus abdominis (deep)	Intercostal	Branch
				Bulbospongiosus	Pudendal	Cord
				Sphinctor ani externus (superficial and deep portions)	Pudendal	Cord
				Levator ani (puborectal portion), or (anterior portion, anterior fibers of the deep pelvic layer)	Pudendal	Cord
		14	Internal oblique	Intercostales interni	Intercostal	Branch
				Abdominal internal oblique	Intercostal, Ilioinguinal	Branch
				Cremaster	Genitofemoral	Branch
				Ischiocavernosus	Pudendal	Branch
				Superficial transverse perineal	Pudendal	Branch
				Levator ani (anterior portion, superficial perineal layer	Pudendal	Branch
		15	Transversus	Intercostales intimi	Intercostal	Branch
				Subcostalis	Nerve to	Branch
				Transverse thoracis	Intercostal	Branch
				Transversus abdominis	Ilioinguinal	Branch
				Deep transverse perineal	Pudendal	Branch
				Levator ani (posterior portion)	Pudendal	Branch
		16	Longus-Transversus	Longus capitis	Nerve to	Root
				Longus colli	Nerve to	Root
				Rectus capitis ant.	Nerve to	Root
				Scalenus anterior	Nerve to	Root
				Levator ani (anterior portion, posterior fibers of the deep pelvic layer)	Nerve to	Root
		17	Deep costal	Tranversarii (anterior set)	Nerve to	Root
				Rectus capitis ant.	Nerve to	Root

Supplementary Table 1 Classification of human muscles in the three component model.

			Class	Muscle	Nerve	Origin
Dorsal rami	Medial subgroup	1	Submultifidus	Rectus capitis post.	Nerve to	Root
				Obliquus capitis inferior	Nerve to	Root
				Rotatores	Nerve to	Root
		2	Multifidus	Multifidus	Nerve to	Root
				Semispinalis	Nerve to	Root
	Lateral subgroup	3	Longissimus	Obliquus capitis superior	Nerve to	Root
				Longissimus	Nerve to	Root
				Splenius	Nerve to	Root
		4	Iliocostalis	Iliocostalis	Nerve to	Root
Intermediate rami		5	Intertransversarii	Intertransversarii proper (medial slip, of the posterior set)	Nerve to	Root
				Thoracic intertransversarii (medial half, medial slip of the posterior set)	Nerve to	Root
				Levator costarum (lateral half, medial slip of the posterior set, thoracic intertransversarii)	Nerve to	Root
Ventral rami	Dorsal subgroup	6	Supracostal	Serratus posterior superior	Superfical intercostal	Root
				Serratus posterior inferior	Superfical intercostal	Root
		7	External Oblique	Cervical intertranversarii (lateral slip, posterior set)	Nerve to	Root
				Scalenus medius	Nerve to	Root
				Scalenus posterior	Nerve to	Root
				Levator scapulae	Dorsal scapula	Root
				Rhomboid	Dorsal scapula	Root
				Serratus anterior	Long thoracic	Root
				Intercostales externi	Superficial intercostal	Root
				Lumbar intertransversarii (lateral slip, posterior set)	Nerve to	Root
				Quadratus lumborum	Nerve to	Root
				Coccygeal	Nerves to	Root
		8	Dorsal extramural	Teres minor	Axillary	Cord
				Gluteus medius	Superior gluteal	Division
				Gluteus minimus	Superior gluteal	Division
				Piriformis	Nerves to	Division

Supplementary Table 1 Classification of human muscles in the three component model.

			Class	Muscle	Nerve	Origin
Ventral rami	Dorsal subgroup	9	Limb extensor	Deltoid	Axillary	Cord
				Latissimus dorsi	Thoracodorsal	Cord
				Subscapularis	Subscapularis	Cord
				Teres major	Subscapularis	Cord
				Gluteus maximus	Inferior gluteal	DIvision
				Tensor fascia lata	Superior gluteal	Division
				Extensor muscles in the limb	Plexus	Terminal branch
	Ventral subgroup	10	Limb flexor	Supinatus	Suprascapular	Trunk
				Pectoralis major	Pectoral	Cord
				Posas	Nerve to	Division
				Quadratus femoris	Nerve to	Division
				Obturator internus	Nerve to	Division
				Obturator externus	Obturator	Branch
				Flexor muslces in the limb	Plexus	Terminal branch
		11	Ventral extramural	Subclavius	Subclavius	Trunk
				Pectoralis minor	Pectoral	Cord
				Abdominal external oblique	Intercostal (lateral cutaneous branch)	Cord
				Iliacus	Nerve to	Division
				Gemellus (inferior)	Nerve to	Division
				Gemellus (superior)	Nerve to	Division
		12	Rectus (superficial)	Infrahyoid (superficial)	Ansa cervicalis	Root
				Rectus abdominus (superficial)	Intercostal	Root
				Sphinctor ani externus (subcutaneous portion)	Nerve to	Root
		13	Rectus (deep)	Infrahyoid (deep)	Ansa cervicalis	Branch
				Rectus abdominis (deep)	Intercostal	Branch
				Bulbospongiosus	Pudendal	Cord
				Sphinctor ani externus (superficial and deep portions)	Pudendal	Cord
				Levator ani (puborectal portion), or (anterior portion, anterior fibers of the deep pelvic layer)	Pudendal	Cord
		14	Internal oblique	Intercostales interni	Intercostal	Branch
				Abdominal internal oblique	Intercostal, Ilioinguinal	Branch
				Cremaster	Genitofemoral	Branch
				Ischiocavernosus	Pudendal	Branch
				Superficial transverse perineal	Pudendal	Branch
				Levator ani (anterior portion, superficial perineal layer	Pudendal	Branch

Supplementary Table 1   Classification of human muscles in the three component model.

			Class	Muscle	Nerve	Origin
Ventral rami	Ventral subgroup	15	Transversus	Intercostales intimi	Intercostal	Branch
				Subcostalis	Nerve to	Branch
				Transverse thoracis	Intercostal	Branch
				Transversus abdominis	Ilioinguinal	Branch
				Deep transverse perineal	Pudendal	Branch
				Levator ani (posterior portion)	Pudendal	Branch
		16	Longus-Transversus	Longus capitis	Nerve to	Root
				Longus colli	Nerve to	Root
				Rectus capitis ant.	Nerve to	Root
				Scalenus anterior	Nerve to	Root
				Levator ani (anterior portion, posterior fibers of the deep pelvic layer)	Nerve to	Root
		17	Deep costal	Tranversarii (anterior set)	Nerve to	Root
				Rectus capitis ant.	Nerve to	Root

Supplementary Table 2 Allocation of pelvic floor muscles into the muscle classes in our model.

Wu et al., 2015		Deep transverse perineal	Superficial transverse perineal<br>Ischiocavernosum	Bulbospongiosum				Perineal muscles
Plochocki et al., 2016			Superficial transverse perineal<br>Ischiocavernosum	Bulbospongiosum				
Baramee et al., 2020			Superficial transverse perineal<br>Ischiocavernosum	Bulbospongiosum				
Oh and Kark, 1972				Deep EAS<br>Puborectalis	Superficial EAS	Subcutaneous EAS		External anal sphinctor (EAS) muscle
Al-Ali et al., 2009				Upper part		Lower part		
Wu et al., 2015				Deep puborectalis	Superficial puborectalis	EAS proper		
Plochocki et al., 2016				Deep EAS	Superficial EAS	Subcutaneous EAS		
	**Longus-transverse**	**Transverse**	**Internal oblique**	**Deep rectus 2**	**Deep rectus 1**	**Superficial rectus 2**	**Superficial rectus 1**	**Muscle class**
Ayoub, 1979		Posterior portion	Anterior portion, pelvic layer	Anterior portion, middle layer	Anterior portion, perineal layer			Levator ani muscle
Bustami, 1989	Anterior portion, posterior fibers of the superficial perineal layer	Posterior portion	Anterior portion, posterior fibers of the deep pelvic layer	Anterior portion, anterior fibers of the deep pelvic layer	Anterior portion, anterior fibers of the superficial perineal layer			
Wu et al., 2015		Medial pubovicelaris	Lateral pubovicelaris					

References in Supplementary Information

Al-Ali, S., Blyth, P., Beatty, S., Duang, A., Parry, B., and Bissett, I.P. (2009). Correlation between gross anatomical topography, sectional sheet plastination, microscopic anatomy and endoanal sonography of the anal sphincter complex in human males. J. Anat. 215(2), 212–220.

Arakawa, T., Hayashi, S., Kinugasa, Y., Murakami, G., and Fujimiya, M. (2010). Development of the external anal sphincter with special reference to intergender difference: Observations of mid-term fetuses (15-30 weeks of gestation). Okajimas Folia Anatomica Japonica 87(2), 49–58.

Ayoub, S.F. (1979). The anterior fibers of the levator ani muscle in man. J Anat. 128 (Pt 3), 571-580.

Baramee, P., Muro, S., Suriyut, J., Harada, M., and Akita, K. (2020). Three muscle slings of the pelvic floor in women: An anatomic study. Anat. Sci. Int. 95(1), 47-53.

Bustami, F.M. (1988). A reappraisal of the anatomy of the levator ani muscle in man. Acta. Morphol. Neer. SC. 26(4), 255–268.

Durland, J.L., Sferlazzo, M., Logan, M., and Burke, A.C. (2008). Visualizing the lateral somitic frontier in the Prx1Cre transgenic mouse. J. Anat. 212(5), 590–602

Fritsch, H., Brenner, E., Lienemann, A., and Ludwikowski, B. (2002). Anal sphincter complex: Reinterpreted morphology and its clinical relevance. Dis. Colon Rectum 45(2), 188–194.

Fröhlich, B., Hötzinger, H., and Fritsch, H. (1997). Tomographical anatomy of the pelvis, pelvic floor, and related structures. Clin. Anat. 10(4), 223–230.

Kodama K. (1986). Morphological significance of the supracostal muscles, and the superficial intercostal nerve--a new definition. Acta Anat. Nippon. 61(2), 107-129 (in Japanese, abstract and figure legends in English).

Nishi, S. (1938). “Muskeln des Rumpfes,” in Handbuch der Vergleichenden Anatomie der Wirbeltiere, Bd 5, eds. L. Bolk, E. Göppert, E. Kallius, and W. Lubosch (Berlin: Urban and Schwarzenberg), 351–446.

Oh, C., and Kark, A. E. (1972). Anatomy of the external anal sphincter. Brit. J. Surg. 59(9), 717–723.

Plochocki, J.H., Rodriguez-Sosa, J.R., Adrian, B., Ruiz, S.A., and Hall, M. I. (2016). A functional and clinical reinterpretation of human perineal neuromuscular anatomy: Application to sexual function and continence: Human Perineal Anatomy. Clin. Anat. 29(8), 1053–1058.

Rousso, D.L., Gaber, Z.B., Wellik, D., Morrisey, E.E., and Novitch, B.G. (2008). Coordinated actions of the forkhead protein Foxp1 and Hox proteins in the columnar organization of spinal motor neurons. Neuron 59(2), 226–240.

Sato, T. (1968). Morphological study on the levatores costarum and the intertransversarius muscles. Acta Anat. Nippon. 43(5), 305-325 (in Japanese, abstract and figure legends in English).

Sato, T. (1971) Innervation of the levatores costarum in man-morphological notes on the paravertebral muscles. Acta Anat. Nippon. 46(3), 172-192 (in Japanese, abstract and figure legends in English).

Sato, T. (1974). On the rami intermedii of the spinal nerves and their equivalent offshoots. A contribution to classification of the trunk muscles. Zeitschrift Fur Anatomie Und Entwicklungsgeschichte 143(2), 143–157.

Shafik, A. (1975). New concept of the anatomy of the anal sphincter mechanism and the physiology of defecation. II. Anatomy of the levator ani muscle with special reference to puborectalis. Invest. Urol. 13(3), 175–182.

Standring, S. (2015). Gray's Anatomy: The Anatomical Basis of Clinical Practice. 41 edition. Elsevier.

Suriyut, J., Muro, S., Baramee, P., Harada, M., and Akita, K. (2020). Various significant connections of the male pelvic floor muscles with special reference to the anal and urethral sphincter muscles. Anat. Sci. Int. 95(3), 305–312.

Thompson, P. (1899). On the Levator Ani, or Ischio-Anal Muscle of Ungulates, with Special Reference to its Morphology. J. Anat. Physiol. 33(Pt 3), 423–433.

Wu, Y., Dabhoiwala, N.F., Hagoort, J., Shan, J.-L., Tan, L.-W., Fang, B.-J., Zhang, S.-X., and Lamers, W.H. (2015). 3D Topography of the Young Adult Anal Sphincter Complex Reconstructed from Undeformed Serial Anatomical Sections. PLOS ONE, 10(8), e0132226.

Supplementary Figure and Legend

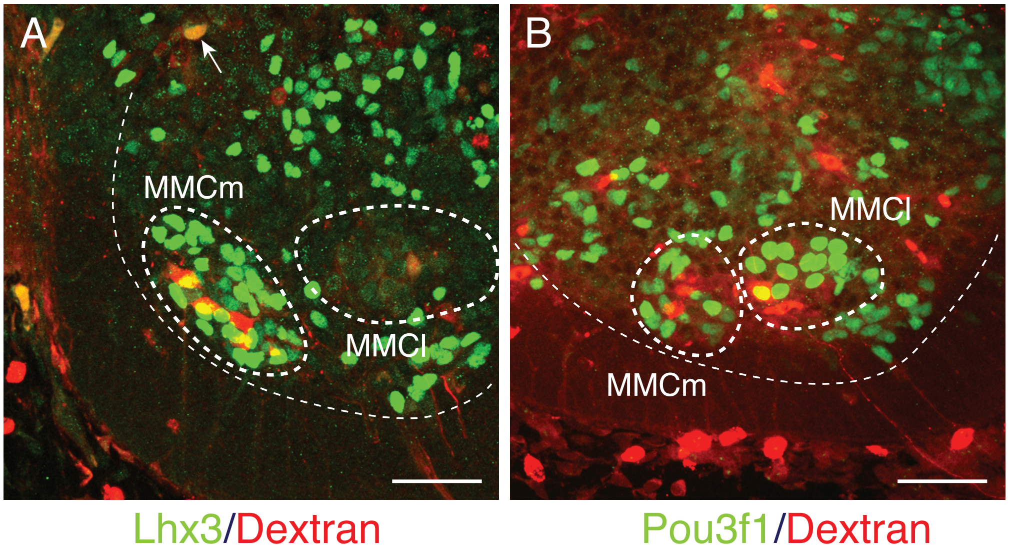

Supplementary Figure. Lhx3 and Pou3f1 expressions in motor neurons innervating the infrahyoid muscles.

All micrographs are transverse sections of the upper cervical spinal cord (C1 and 2) from E13 mouse embryos. The midline of the spinal cord is toward the left side of the micrograph, and the dorsal side is toward the upper part. The thin dashed lines represent the boundaries of the ventral horns. The thick dashed lines encircle MMCm and MMCl. The labeled MNs are Lhx3-positive in the MMCm in (A) or Pou3f1-positive in the MMCl in (B). The arrow in (A) indicates a labeled hypoglossal MN. Scale bars are 100 μm.
